# Molecular beacons with oxidized bases report on substrate specificity of DNA oxoguanine glycosylases†

**DOI:** 10.1039/d1sc05648d

**Published:** 2022-02-16

**Authors:** Jingjing Sun, Nicole M. Antczak, Hailey L. Gahlon, Shana J. Sturla

**Affiliations:** Department of Health Sciences and Technology, ETH Zürich Zürich 8092 Switzerland sturlas@ethz.ch; Department of Biological Engineering, Massachusetts Institute of Technology 77 Massachusetts Avenue Cambridge MA 02139 USA; Department of Chemistry, Skidmore College 815 North Broadway Saratoga Springs NY 12866 USA

## Abstract

DNA glycosylase enzymes recognize and remove structurally distinct modified forms of DNA bases, thereby repairing genomic DNA from chemically induced damage or erasing epigenetic marks. However, these enzymes are often promiscuous, and advanced tools are needed to evaluate and engineer their substrate specificity. Thus, in the present study, we developed a new strategy to rapidly profile the substrate specificity of 8-oxoguanine glycosylases, which cleave biologically relevant oxidized forms of guanine. We monitored the enzymatic excision of fluorophore-labeled oligonucleotides containing synthetic modifications 8-oxoG and FapyG, or G. Using this molecular beacon approach, we identified several hOGG1 mutants with higher specificity for FapyG than 8-oxoG. This approach and the newly synthesized probes will be useful for the characterization of glycosylase substrate specificity and damage excision mechanisms, as well as for evaluating engineered enzymes with altered reactivities.

## Abstract 2

A three-color fluorescent molecular beacon assay for rapid profiling of substrate specificity of hOGG1 variants, and for engineering proteins to map genomic modifications.

## Graphical abstract

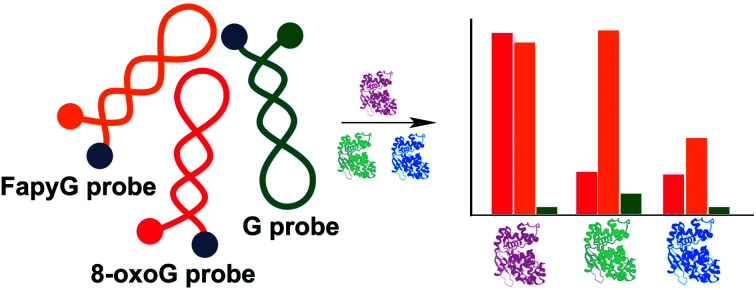

## Introduction

DNA is constantly altered by epigenetic modifications and chemical exposures. Sophisticated molecular mechanisms involving epigenetic writers/erasers and repair enzymes have evolved, therefore, to manipulate such structures.^1,2^ Relying on many of these enzymes, modern DNA manipulation and gene editing technologies are transforming our understanding of the genome and how to treat diseases, such as involving zinc-finger nucleases,^3^ transcription activator-like effector nucleases (TALENS),^4^ clustered regularly interspaced short palindromic repeat (CRISPR)^5^ systems, base editors,^6^ and glycosylases.^7^ However, many of these enzymes are inherently promiscuous, limiting their specificity to experimentally manipulate DNA on the basis of particular structural modifications in DNA.^8,9^ For example, DNA glycosylases are efficient base-excision enzymes that remove nucleobases with diverse small modifications, such as those resulting from oxidation and methylation of DNA. Thus, new chemical and enzymological strategies for defining and altering the substrate specificity of glycosylases are expected to advance precision DNA manipulation after recognizing chemical modifications.

**Fig. 1:**
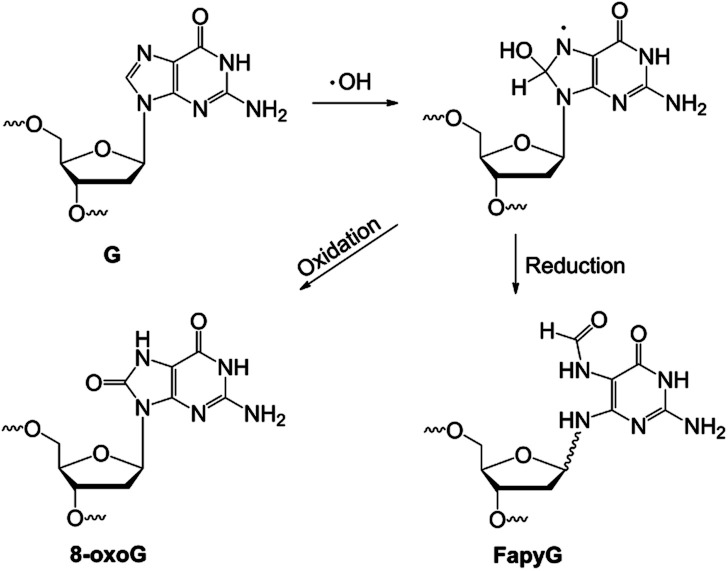
Formation of 8-oxoG and FapyG.

DNA base oxidation, arising from reactions with reactive oxygen species (ROS) resulting from environmental chemical exposures, UV or ionizing irradiation, and cellular metabolism,^10^ is a major form of DNA chemical modification, significantly impacting genome integrity and cell function.^11^ Of the canonical bases in DNA, guanine is the most easily oxidized, due to its low redox potential amongst the four DNA bases.^12^ Several structurally distinct oxidation products result with potentially distinct genomic distribution and biological impacts.^13^ Major products include 8-oxoguanine (8-oxoG) and 2,6-diamino-4-oxo-5-formamidopyrimidine (FapyG) (Fig. 1).^14^ Error-prone replication of 8-oxoG or FapyG induces G to T transversion and G to A transition mutations.^15,16^ DNA glycosylases that remove these oxidized nucleobases initiate base excision repair,^13^ and are key reagents for emerging DNA damage detection and sequencing technologies.^7,17–22^

Oxoguanine glycosylases have been used widely to detect DNA oxidation, including in the comet assay,^19^ mass spectrometry^20^ and DNA damage sequencing.^18,21,22^ In the oxidation-sequencing methods click-code-seq and entrap-seq, formamidopyrimidine-DNA glycosylase (Fpg) or a human 8-oxoG glycosylase (hOGG1) K249Q mutant were used to enrich and map guanine oxidation in yeast and mouse genomes, respectively.^18,22^ While the majority of mapped sites were expected to be 8-oxoG due to its high prevalence in the genome, the general approach is actually limited to mapping the oxidized base substrate scope of the glycosylase used, and the distribution of specific chemical forms of oxidation products could not be resolved, such as distinguishing between 8-oxoG *vs.* FapyG. As a result, there are no high specificity DNA oxidation maps available, and there is a need to rapidly assess the substrate specificity of enzyme variants in order to engineer enzymes with substrate scopes different from what is found in nature.

The most common way to characterize glycosylase activity is separating and imaging cleaved DNA by gel electrophoresis,^23^ a method with insufficient throughput for screening enzyme variants. Likewise, LC-MS and qPCR can be used but involve tedious sample preparation.^24,25^ Fluorescent molecular beacons have been established as excellent tools for characterizing glycosylase activity in a simple and real-time manner.^26–29^ However, to our knowledge, such a strategy has not been established as yet to evaluate glycosylase substrate specificity and screen mutants with altered function due to a lack of synthetic probes containing different glycosylase substrates and the typical use of purified glycosylase enzymes.

**Fig. 2:**
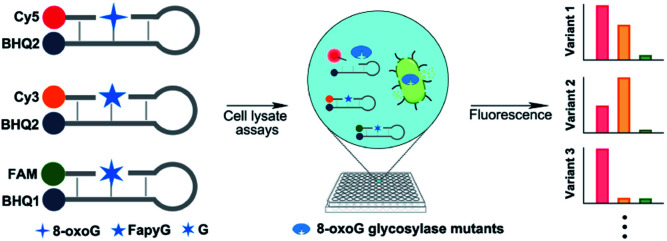
Illustration of the 8-oxoG glycosylase-screening platform.

In this study, we developed a three-color molecular beacon system to evaluate the 8-oxoG *vs.* FapyG specificity of glycosylase enzymes. The goal was to compare enzyme activity using oligonucleotide substrates containing 8-oxoG or FapyG, or a control oligonucleotide containing G (Fig. 2). Three molecular beacons were created for this platform, each with a different fluorophore that is released as an indicator of the relative efficiency of glycosylase activity on the corresponding base structure. This novel molecular beacon platform was used to characterize the specificity of hOGG1 and several hOGG1 variants in crude whole-cell lysates. Furthermore, we evaluated the substrate specificity of a random hOGG1 D322 variant library and identified variants with an increased relative capacity to excise FapyG. This approach has the capacity to report on glycosylase substrate specificity directly in cell lysates by using orthogonal fluorescent reporters, offering the possibility for high-throughput glycosylase variant screening.

## Results and discussion

### Synthesis of three-color molecular beacons

**Scheme 1:**
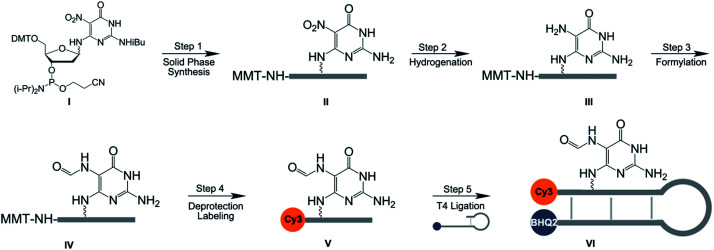
Synthesis of a FapyG-containing molecular beacon. Step 1: standard solid phase synthesis, DMT-on mode; step 2: NaBH_4_, nickel boride, room temperature, 10 min; step 3: *N*-formylimidazole, THF, room temperature, 2 h; step 4: 80% acetic acid in water, room temperature, 20 min; cyanine 3-NHS ester, DMSO, room temperature, 3 h; step 5: T4 ligase, DTT-free buffer, room temperature, 2 h. Cy3: cyanine 3; BHQ2: black hole quencher 2.

As a first step to create the three-color molecular beacon system to evaluate the specificity of glycosylase enzymes for 8-oxoG *vs.* FapyG excision, we synthesized molecular beacons containing competing substrates at a defined position, and also equipped them with a fluorophore and quencher pair. We employed a post-synthetic double modification strategy whereby a nitro-pyrimidine (NP-dG) used previously as a FapyG precursor^30^ (Schemes 1I; S1 and S6†) and a 5′ amino guanosine (AM-dG) phosphoramidite (Schemes S2 and S10†), were synthesized and incorporated into the 10mer oligonucleotide 5′-AM-dGGTCTNP-dGATGG-3′ (Scheme 1II). We found that using AM-dG^31^ for the attachment of FAM provided an ∼80-fold higher signal-to-noise ratio than using a commercial C6 amino modifier (Fig. S1†). Initial attempts to catalyze the reduction of the nitro group (Scheme 1, step 2) with palladium on carbon^30^ lead to the cleavage of oligonucleotides at the NP-dG site and very low product yields; this problem was circumvented by using NaBH_4_ and a nickel boride catalyst (Fig. S2†).^32^ Subsequent steps included deprotection of the MMT group with 80% acetic acid and installation of the Cy3 fluorophore on the 5′ end of the oligonucleotide by NHS ester chemistry (Scheme 1, step 4). Finally, the full-length molecular beacon was obtained by ligating the FapyG-containing 10mer oligonucleotide with a BHQ2-modified oligonucleotide (Scheme 1, step 5; Fig. S3†) and purification on a polyacrylamide gel. As in previous studies involving a Fapy-containing oligonucleotide, the modification exists as a mixture of α and β anomers.^33–36^ Finally, 8-oxoG- and G-containing molecular beacons were synthesized in the same manner as FapyG (experimental section).

### Optimization and validation of molecular beacon-based assay

**Fig. 3:**
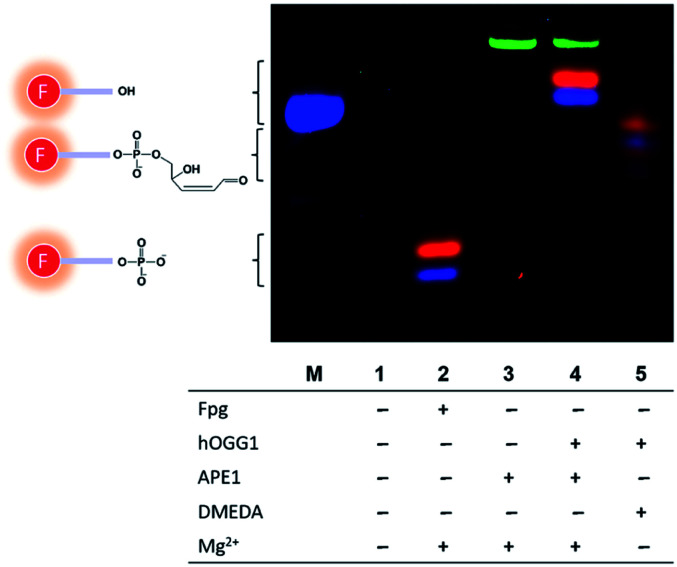
PAGE analysis of glycosylase-mediated cleavage of the molecular beacons (lanes 1–5). The green, blue and red bands indicate the cleavage products of MB-FAM-G, MB-Cy3-FapyG and MB-Cy5-8oxoG, respectively. Lane M indicates a Cy3-labeled 5mer marker.

Having color-coded 8-oxoG, FapyG and G molecular beacon substrates in hand, we tested the relative cleavage rates in the presence of enzymes: Fpg, hOGG1, and apurinic/apyrimidinic endonuclease (APE1). Fpg efficiently cleaved the molecular beacons containing FapyG (Cy3) and 8-oxoG (Cy5), yielding Cy3- and Cy5-labeled 3′-phosphated short oligonucleotides (Fig. 3, lane 2). Reactions catalyzed by hOGG1 and APE1 yielded 3′-hydroxyl oligonucleotides (Fig. 3, lane 4). The identity of the 5mer products arising from the cleavage catalyzed by Fpg (Fig. 3, lane 2) or hOGG1/APE1 (Fig. 3, lane 4) was assigned on the basis of MS confirmation of analogous structures arising from the cleavage of the corresponding C6 amino-modified fragments identified in initial experiments with MB-Cy5(C6)-8oxoG and duplex-Cy5-8oxoG (Fig. S4†). The positive charge of Cy3/Cy5 appeared to impede the migration of the 5mer oligonucleotide (Fig. 3, lane 4; Fig. S5†), causing gel shifts slower than expected.^37^ Finally, with APE1 alone or hOGG1 plus APE1, a FAM signal was also observed from the cleavage of the unmodified molecular beacon (MB-FAM-G) (Fig. 3, lanes 3 and 4; Fig. S6†). By evaluating the reaction products by mass spectrometry, we determined that the increase in the FAM signal was caused by the cleavage of the BHQ1 quencher at the 3′ end of the molecular beacon (Fig. S7†), possibly due to the 3′-diesterase activity of APE1. This effect was observed for BHQ1 but not for BHQ2.^38,39^ To avoid quencher cleavage, *N*,*N*-dimethylethylenediamine (DMEDA), which was demonstrated previously to promote abasic site cleavage,^40^ was used in place of APE1. Replacing APE1 with DMEDA in the hOGG1 excision reaction also was effective for cleaving 8-oxoG- and FapyG-containing molecular beacons, producing mainly β-elimination products (Fig. 3, lane 5).^40^ Furthermore, a linear correlation was observed between the fluorescence signal resulting from Cy5 and the concentration of the molecular beacon containing 8-oxoG (concentration range 50–500 nM, *R*^2^ = 0.994, Fig. S8†). The outcome of these studies was an optimized robust strategy to compare the specificity of purified glycosylases for the cleavage of 8-oxoG *vs.* FapyG.

### Glycosylase activity evaluation directly in crude cell lysates

**Fig. 4:**
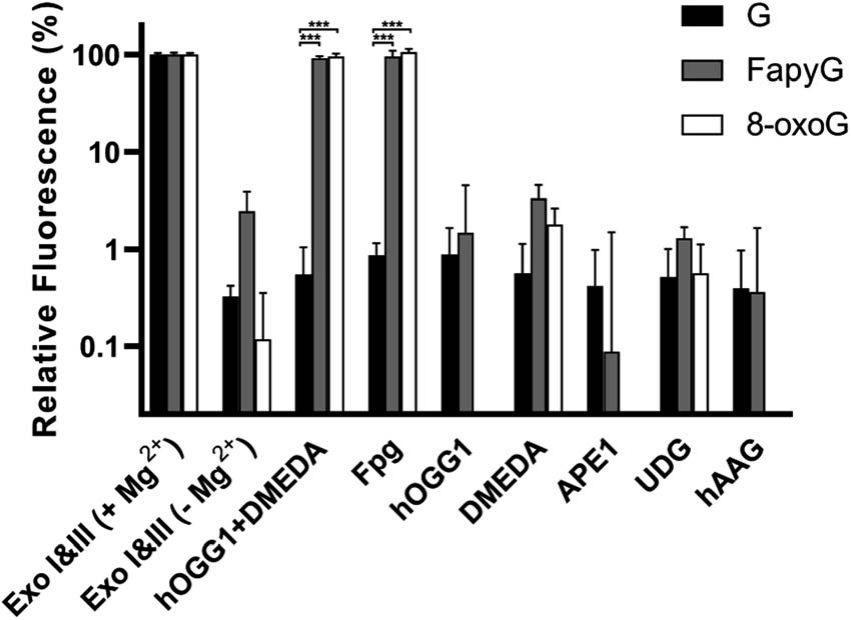
Response of the three molecular beacons to glycosylases. Exo I & III were used in Mg^2+^-containing buffer as a positive control. All the other reactions were performed in Mg^2+^-free buffer. The *Y* axis is on the log_10_ scale. *P* values were determined by one-way ANOVA. *** indicates *P* < 0.001, *n* = 4.

For rapid profiling of several glycosylase variants, such as those generated in the course of protein engineering, the strategy should also effectively report on enzyme specificity from cell lysates rather than purified enzymes. Therefore, EDTA was added to the mixture to sequester magnesium and inhibit exonuclease (Exo I & III) activity without reducing hOGG1 or Fpg function (Fig. 4). Meanwhile, less than 3% cleavage was observed in assays with hOGG1, DMEDA, APE1, uracil-DNA glycosylase (UDG) or human alkyl adenine DNA glycosylase (hAAG). Furthermore, we transformed a hOGG1 plasmid into *E. coli* cells and overexpressed hOGG1. Direct analysis of these cell lysates led to ∼80% cleavage of the molecular beacons within 30 min, compared to 2–5% cleavage by lysate from cells transformed with an empty vector (Fig. S9†). Thus, we could evaluate the glycosylase specificity in *E. coli* cell lysates without protein purification.

### Substrate specificity profiling of known hOGG1 variants and single mutation variant libraries

**Fig. 5:**
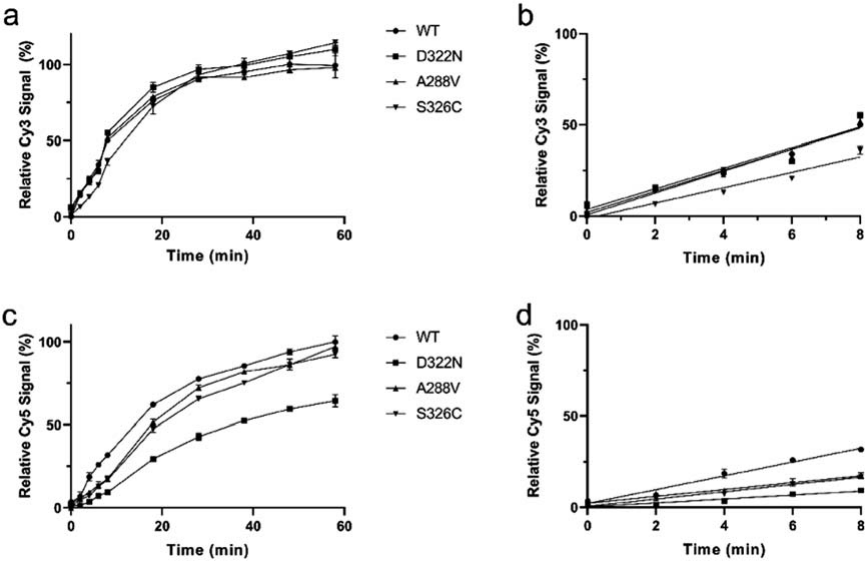
Enzymatic excision kinetics for hOGG1 single nucleotide variants (D322N, A288V and S326C). (a) Increase in Cy3 signal corresponding to FapyG cleavage over 60 min incubation. (b) Initial differences in first 8 min amongst enzyme variants in Cy3 signal. (c) Increase in Cy5 signal corresponding to 8-oxoG cleavage over 60 min incubation. (d) Initial differences in first 8 min amongst enzyme variants in Cy5 signal. The signal from WT hOGG1 reactions after 1 h were set to 100%. The data in (b and d), from first 8 min, were used for linear regression to determine relative reaction rates. *n* = 4.

**Fig. 6:**
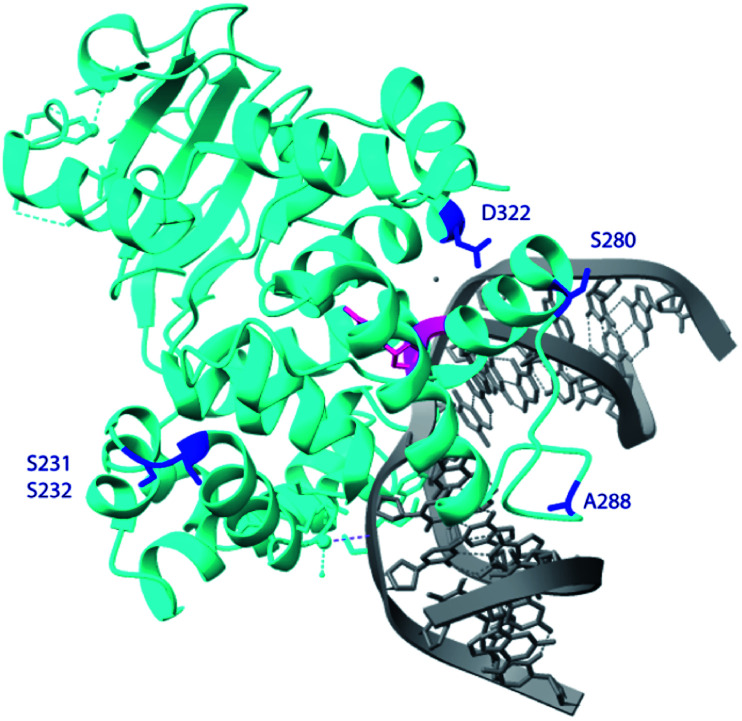
Crystal structure of hOGG1 (PDB ID 6W0M).^43^ The residues of interest are highlighted in blue, except S326 which is not in the crystal structure, 8-oxoG is in pink and the remainder of the DNA in grey.

With a robust *in situ* glycosylase profiling assay established, we set out to characterize the influence of particular amino acid residues in the substrate specificity of hOGG1. Thus, hOGG1 single-nucleotide polymorphic variants (A288V, D322N and S326C) and phosphorylation mimics (S231E, S232E, S280E and S326E), previously characterized by using traditional gel-based assays,^23,41,42^ were profiled with regards to relative initial rates of enzymatic excision (Fig. 5 and S10†) and substrate specificities (Table 1) using the molecular beacon platform. Variants with mutations more distal to the catalytic center (S326C, S326E, S231E, S232E and S280E) had similar FapyG/8-oxoG specificity ratios to the wild type enzyme, ranging between 0.85 and 1.33-fold (Table 1 and Fig. 6). However, the D322N mutant, altered close to the catalytic center, had the highest substrate specificity (3.3-fold) for FapyG. These results demonstrated the simplicity and robustness of the molecular beacon strategy and suggested a candidate residue for further tuning glycosylase specificity.

**Table 1:** Relative initial rates of FapyG *vs.* 8-oxoG excision by hOGG1 variants^a^

Variants	FapyG	8-oxoG	FapyG/8-oxoG
D322N	0.94 ± 0.16	0.28 ± 0.05	3.3 ± 0.57
A288V	0.96 ± 0.10	0.50 ± 0.02	1.94 ± 0.15
S326C	0.70 ± 0.10	0.53 ± 0.04	1.33 ± 0.15
S326E	0.81 ± 0.05	0.82 ± 0.03	0.99 ± 0.05
S231E	1.04 ± 0.19	0.91 ± 0.09	1.14 ± 0.16
S232E	0.96 ± 0.14	0.80 ± 0.06	1.20 ± 0.14
S280E	1.68 ± 0.07	1.98 ± 0.11	0.85 ± 0.05

a Values are initial rates relative to wild type hOGG1, and were calculated on the basis of change in fluorescence emission intensity per min during the first 8 min of reaction. Data are in Fig. 5 and S10.

**Fig. 7:**
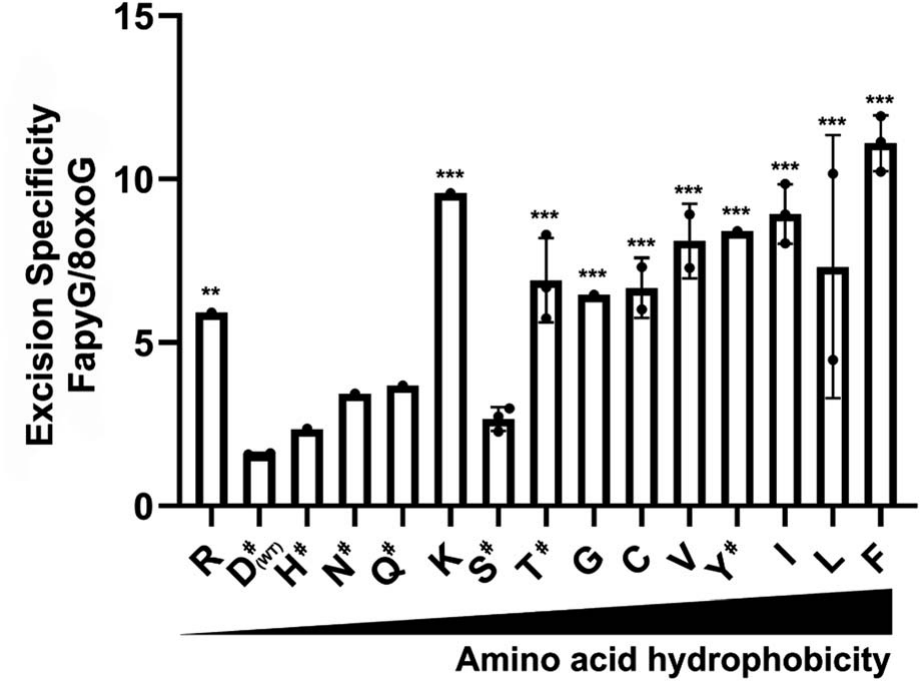
Excision specificity of hOGG1 D322 variants for FapyG over 8-oxoG. The *Y* axis indicates fold changes of FapyG excision *vs.* 8-oxoG excision. The *X*-axis indicates different amino acids at the D322 site in increasing order of amino acid side chain hydrophobicity. # indicates amino acids having a hydrogen acceptor. Each dot indicates a single colony (*n* = 4). The comparison was relative to wild type (WT). *P* values were determined by one-way ANOVA. ** indicates *P* < 0.01, *** indicates *P* < 0.001.

In the crystal structure of hOGG1,^43^ either in the apo form or bound to 8-oxoG, the carboxyl group of D322 forms a hydrogen bond with the imidazole ring of H270. In particular, H270 forms a hydrogen bond with C8=O of the extruded 8-oxoG, serving as a crucial residue during 8-oxoG recognition and repair.^43,44^ Thus, we profiled the specificity of a hOGG1 D322 variant library derived from 48 colonies generated *via* site-directed mutagenesis, harbouring 14 different single mutations at site 322 (Fig. 7). Variants D322H, D322S, D322N and D322Q, which have a hydrogen bond acceptor at site 322, had a similar specificity to wild type hOGG1. On the other hand, variants D322R, D322C, D322G, D322L, D322V, D322I, D322K and D322F, which lack hydrogen-bond-acceptor capacity were more than 4-fold selective for FapyG over 8-oxoG. Similar specificity was observed for D322T, which has an amino and hydroxyl group, and D322Y that has an aromatic side chain. The truncated protein (residues 1–321) and D322P mutant did not excise either FapyG or 8-oxoG (Fig. S11†). Proline (P) is known to perturb the α helix structure, and therefore it may change the global structure of hOGG1, leading to the observed loss of activity.^45^ In general, hOGG1 variants containing hydrophobic amino acids at site 322 appear to have better excision specificity for FapyG over 8-oxoG than hydrophilic amino acids, except for positively charged arginine (R) and lysine (K) (Fig. 7). A caution in interpreting the specificity data is potential conformational effects on FapyG cleavage, since it exists as equilibrating anomers.^33–36^ Finally, these results suggest that the properties of the amino acid residue at position 322, including hydrogen donor/acceptor, hydrophobicity, stereochemistry, side chain size and charge state, influence the substrate specificity of hOGG1. As the crystal structure of hOGG1 bound to FapyG has not been elucidated, further studies are needed to clarify the role of D322 in the excision of FapyG as compared to 8-oxoG.

C253 is another important residue for 8-oxoG cleavage by hOOG1. It forms a dipole–dipole interaction with the K249 residue to sandwich the extruded 8-oxoG.^46,47^ Therefore, we also generated several C253 hOGG1 variants and evaluated their substrate selectivity (Fig. S11†) in the same fashion as described for D322 variants (Fig. 7). Mutation of C253 led to loss of hOGG1 activity for excision of 8-oxoG, suggesting that the presence of aliphatic residues at site 253 of hOGG1 blocks the entrance of 8-oxoG, consistent with previous observations of low activity of variant C253L.^48^ On the other hand, mutants C253M and C253L retained activity for FapyG excision, with more than 10-fold specificity for FapyG over 8-oxoG. These results suggest that a large residue at site 253 significantly impedes 8-oxoG excision, but has little effect on FapyG excision, potentially due to its greater structural flexibility. These observations suggest that despite the similarities of 8-oxoG and FapyG, there are features of their interactions with glycosylases that can allow them to be distinguished. Nonetheless, extensive further screening of other single- or multi-mutation variants will be required for very large gains in substrate selectivity. Finally, the results from screening D322 and C253 mutants demonstrate the potency of the molecular beacon method for this purpose.

## Conclusions

In conclusion, we developed a three-color molecular beacon platform that can be used to evaluate the substrate specificity of 8-oxoG glycosylases for excision of 8-oxoG *vs.* FapyG. The approach was optimized to profile *E. coli* cell lysates overexpressing hOGG1, thus providing a simple and rapid assay for screening glycosylase activity and specificity that does not require protein purification. Using this approach, we identified several D322 and C253 variants with higher FapyG/8-oxoG specificity than the wild type enzyme. These residues appear to help stabilize the interaction between hOGG1 and the oxidized base. It is anticipated that the strategy and new synthetic molecular beacons reported here will enable further development and understanding of 8-oxoG glycosylase function and substrate scope. Furthermore, the simple and modular strategy for rapidly profiling glycosylase substrate specificity may be used with any modifications incorporated into the beacons to understand and engineer the specificity of diverse DNA-cleaving enzymes.

## Experimental

### Materials and reagents

Solvents and chemical reagents were purchased from Sigma Aldrich if not specifically mentioned otherwise. Commercial enzymes T4 ligase, exonuclease I, exonuclease III, Fpg, APE1, hAAG and UDG were purchased from New England Biolabs and hOGG1 was purchased from R&D system.

### Synthesis of oligonucleotides

Oligonucleotides were synthesized on a MerMade 4 DNA/RNA synthesizer (BioAutomation Corporation, USA) with reagents from Glen Research (USA). Oligonucleotides containing 8-oxoguanine and guanine were synthesized in DMT-off mode and oligonucleotides containing the nitro-precursor (Scheme 1(II)) were synthesized in DMT-on mode. The subsequent resin cleavage and deprotection was carried out in concentrated ammonium hydroxide at room temperature for 24 h. The deprotection of oligonucleotides containing 8-oxoguanine was conducted with 2-mercaptoethanol (0.25 M) in ammonium hydroxide.

### Post-synthesis of oligonucleotides containing FapyG

Approximately 100 OD_260_ of a mixture of oligodeoxynucleotides containing a nitro-precursor (Scheme 1(II)) was dissolved in 490 μL of water, followed by the addition of 10 μL of triethylamine. 1 mg freshly prepared Ni_2_B nanoparticles^32^ was added to the mixture and stirred at room temperature. A total of 10 mg sodium borohydride was added to the mixture in portions over the course of 10 min, and then the nanoparticles were removed by filtration of the mixture through a 0.2 μm centrifugal filter (VWR). The desired hydrogenated product (Scheme 1(III)) was purified on a Sep-Pak C18 Classic cartridge with 360 mg sorbent (Waters) and eluted using acetonitrile : water (50 : 50). The resulting oligodeoxynucleotide solution was reduced to a volume of 500 μL using a Speed-Vac concentrator (Genevac Ltd, UK). Then 25 μL of 2-mercaptoethanol and 25 μL of triethylamine were added to the mixture, followed by 1 mL of freshly prepared formylimidazole^30,49^ (1 M in THF), which was added in portions over the course of 2 h. When the aminopyrimidine oligodeoxynucleotides had completely disappeared, as confirmed by MS analysis, the reaction mixture was evaporated to remove organic solvents and purified on a Sep-Pak C18 Classic cartridge with 360 mg sorbent.

Subsequently, the purified and lyophilized FapyG-containing oligonucleotides (Scheme 1(IV)) were dissolved in 20 μL 80% acetic acid and shaken at room temperature for 20 min. After removing acetic acid with a Speed-Vac concentrator, the MMT-off oligonucleotides were re-dissolved in ice-cold 200 μL sodium bicarbonate solution (0.1 M in H_2_O). The amount of oligonucleotides was measured using a Nanodrop spectrophotometer 2000 (Thermo Fisher Scientific, USA) and about 8-fold excess of Cy3 NHS ester (10 μg μL^−1^ in DMSO, lumiprobe, USA) was added. The reaction was shaken at room temperature and monitored by MS until the starting material disappeared. The excessive dye was removed using a centrifuge filter (MWCO. 3000, Amicon) and the remaining mixture was purified by high performance liquid chromatography (1260 Infinity, Agilent, USA) using a C18 reverse phase column (4.6 × 250 mm, Phenomenex, USA) with a linear gradient of acetonitrile, 0–40%, for 30 min in 0.05 M triethylammonium acetate (TEAA, pH 7.0). The desired oligonucleotides were lyophilized using a freeze dryer (Labconco Corporation, USA), and quantified using a Nanodrop spectrophotometer and characterized by MS (calc.: 3555, found: 3554).

### Post-fluorescent labelling of oligonucleotides containing 8-oxoguanine and guanine

The deprotected oligonucleotides were re-dissolved in 200 μL sodium bicarbonate solution (0.1 M in H_2_O). The mixture was kept on ice, and about 8-fold excess of dye NHS ester (10 μg μL^−1^ in DMSO, lumiprobe, USA) was added, Cy5 for 8-oxoG and FAM for G. The reaction was shaken at room temperature and monitored by MS until the starting material disappeared. The excessive dye was removed using a centrifuge filter (MWCO. 3000, MilliporeSigma, USA) and the remaining mixture was purified using a C18 reverse phase column (4.6 × 250 mm, Phenomenex, USA) on a high performance liquid chromatograph (1260 Infinity, Agilent, USA) with a linear gradient of acetonitrile, 0–40%, for 30 min in 0.05 M triethylammonium acetate (TEAA, pH 7.0). The desired oligonucleotides were lyophilized and quantified using a Nanodrop spectrophotometer characterized by MS. Oligonucleotides containing 8-oxoG, calc.: 3580, found: 3577; oligonucleotides containing G: calc.: 3454, found: 3454. Deconvolution of multiple charged ESI peaks was performed with MagTran 1.03.^50^

### Assembly of molecular beacons

The purified fluorophore-labelled oligonucleotides were annealed with 1.2-equivalents of quencher-labelled oligonucleotides (Eurogentec, Belgium) in 1× cutsmart buffer (New England Biolabs, USA). The resulting mixture was heated at 70 °C for 5 min and slowly cooled to 4 °C (1 °C per min). ATP (final concentration of 1 mM) and T4 ligase (2000 U) were added and allowed to react at room temperature for 2 h. The crude oligonucleotides were loaded onto 20% denaturing polyacrylamide gels (7 M urea). The gels were cooled to 4 °C during electrophoresis (250 V, 1 h). The desired gel bands were cleaved and extracted in 1× TBE buffer at 4 °C overnight in a dark room. The molecular beacons were filtered using a 0.2 μm centrifuge filter, desalted using an MWCO. 3000 centrifuge filter, quantified using a nanodrop, confirmed by MS (Table S1†) and stored at −20 °C until further use.

### Enzymatic assays with molecular beacons

The fluorescence-based assays were conducted in low-volume 384-well plates (Corning, USA) with 10 μL as the final reaction volume and 3 μL mineral oil layered on top to avoid evaporation. All the molecular beacons were annealed in 1× reaction buffer (50 mM potassium acetate, 20 mM tris-acetate, 1 mM EDTA, 100 μg mL^−1^ BSA, pH 7.9) before use, heating the mixture at 70 °C for 5 min and slowly cooled to 4 °C (1 °C per min). DMEDA was prepared as a 1 M solution (10× stock) in water and adjusted to pH 7.9 by using acetic acid. The molecular beacon probes (0.5 μM) in buffer (50 mM potassium acetate, 20 mM tris-acetate, 100 μg mL^−1^ BSA, 1 mM EDTA, 100 mM DMEDA, pH 7.9) were added to wells and mineral oil was gently added to the top of the probe/buffer mixture and the plates were kept at 37 °C for 5 min. Enzymes exonuclease I (0.01 U), exonuclease III (0.1 U), hOGG1 (0.5 pmol), Fpg (0.25 pmol), APE1 (0.25 pmol), hAAG (0.05 U) or UDG (0.05 U) were added to each well, and fluorescence was monitored with a Tecan infinite 2000 plate reader at 37 °C for 60 min. Monitored excitation and emission wavelengths included the FAM channel: Ex 490 nm/9, Em 520 nm/20; Cy3 channel: Ex 540 nm/9, Em 570 nm/20; Cy5 channel: Ex 640 nm/9, Em 670 nm/20. Fluorescence data was normalized using the following equation: (*F*_i_ − *F*_0_)/(*F*_e_ − F_0_) × 100%. *F*_i_ indicates the fluorescence signal of measured samples. *F*_0_ indicates the fluorescence signal of probe-only negative controls. *F*_e_ indicates the fluorescence signal of probes in the presence of Exo I & III.

### *Escherichia coli* strains and cell culture

Plasmids for mutants (D322N, A288V, S326C, S326E, S280E, S231E, and S232E) were provided by Prof. Dmitry O. Zharkov (Novosibirsk State University). These plasmids were transformed into *E. coli* BL21(DE3) competent cells using manufacturer protocols (New England BioLabs, USA).^29^ Single colonies were cultured overnight at 37 °C in LB broth (100 μg per mL ampicillin) and sequenced (Microsynth, Switzerland). The WT hOGG1 plasmid was constructed from a D322N mutant plasmid using a Q5 site-directed mutagenesis kit (New England BioLabs). PCR primers were designed using NEBaseChanger (New England BioLabs, USA): WTF, 5′-ATTGGCGCAGGTCGGCACTGAAC; WTR, 5′-TCCTCATATGAGGACTCTCGTAGCTGCTGCAG. After the PCR and ligation reaction, the plasmid was transformed into *E. coli* BL21(DE3) competent cells^29^ and the cells were grown on a selection plate containing 100 μg per mL ampicillin. Several colonies were isolated and cultured. Plasmids were isolated from these colonies and sequenced (Microsynth, Switzerland). A WT hOGG1 plasmid confirmed by sequencing was used for further experiments. For protein expression, *E. coli* strains containing WT hOGG1 and mutants were cultured in LB broth (100 μg per mL ampicillin) overnight at 37 °C. The starting culture was diluted with LB broth (100 μg per mL ampicillin) and shaken at 37 °C until the cell density reached OD_600_ 0.4–0.6. Then isopropyl-β-d-thiogalactopyranoside (IPTG, final concentration of 0.2 mM) was added, and incubation was continued at 30 °C for 18 h. The cells were washed twice with 1× PBS buffer and twice with 10% glycerol, and then centrifuged (7000*g* for 20 min at 4 °C). The cell pellets were stored at −80 °C for future use.

### Cell lysate assays

All cell lysates were prepared from single clonal *E. coli* isolates treated with lysozyme (1 mg mL^−1^) and 1× protease inhibitor (Roche, Switzerland) on ice for 30 min. Furthermore, the cell lysate assays were conducted in a similar fashion as with the purified proteins in the enzymatic assays using 10^6^ cells. Lysates were added to a mixture of probes and buffer on ice, and the plate was allowed to equilibrate to 37 °C for 2 min. Fluorescence data were acquired in the same manner as described above under enzymatic assays with molecular beacons (data shown in Fig. 5 and S8–S10†).

### Gel analysis

Enzymatic assays were quenched by adding formamide loading buffer (95% formamide, 20 mM EDTA, 0.01% xylene cyanol, and 0.01% bromophenol blue) in a 1 : 1 ratio. The samples were loaded onto 20% denaturing polyacrylamide gels (7 M urea). The gels were cooled to 4 °C during electrophoresis (250 V, 1 h). The gels were imaged with a ChemiDoc MP System (Bio-Rad, USA) with multichannel fluorescence imaging for Cy5, Cy3 and FAM.

### Construction of site-directed D322 and C253 hOGG1 mutants

The D322 and C253 mutant library was created using a Q5 site-directed mutagenesis kit (New England BioLabs, USA). PCR primers were designed using NEBaseChanger (New England BioLabs, USA): D322F, 5′-ATTGGCGCAGNNNGGCACTGAACAGC; D322R, 5′-CCCGCCATGCTCAGGAGC; C253F, 5′-TCAGGCAGATNNNGTCAGCCACCTTG; C253R, 5′-TGGCCCTAGACAAGCCCC. After the PCR and ligation reaction, the plasmid library was transformed into *E. coli* BL21(DE3) competent cells and the cells were grown on a selection plate containing 100 μg per mL ampicillin. The resulting 96 colonies (48 from D322 and 48 from C253) were isolated and cultured in a deep well 96-well plate (Thermo Fisher Scientific, USA) overnight at 37 °C in LB broth (100 μg per mL ampicillin). The starting culture was diluted with LB broth (100 μg per mL ampicillin) and shaken at 37 °C until the cell density reached OD_600_ 0.4–0.6. Then IPTG (final concentration of 0.2 mM) was added, and incubation was continued at 30 °C for 18 h. The cells were washed twice with 1× PBS buffer and twice with 10% glycerol and then centrifuged at 7000*g* for 20 min at 4 °C. These cells were directly used for fluorescence-based hOGG1 activity assays and sequenced by Sanger sequencing (Microsynth, Switzerland).

## Author contributions

J. S. performed the synthetic and biochemical experiments, analysed the data and drafted the paper. N. A. performed structural modelling studies. J. S., N. A., H. G. and S. S. conceived the research, evaluated the results and contributed to writing the manuscript.

## Conflicts of interest

There are no conflicts to declare.

## Supplementary Material

SC-013-D1SC05648D-s001
