# Supplementary material for: Molecular beacons with oxidized bases report on substrate specificity of DNA oxoguanine glycosylases
Source: Chem Sci. 2022 Feb 16;13(15):4295–302. doi: 10.1039/d1sc05648d (PMC9007065; doi:10.1039/d1sc05648d)

Electronic Supplementary Material (ESI) for Chemical Science.
This journal is © The Royal Society of Chemistry 2022

Supporting information

Molecular beacons with oxidized bases report on substrate specificity of DNA oxoguanine glycosylases

Jingjing Sun[a], [b], Nicole M. Antczak[a], [c], Hailey L. Gahlon[a] and Shana J. Sturla*[a]

[a] Dr. J. Sun, Dr. N. M. Antczak, Dr. H. L. Gahlon and Prof. Dr. S. J. Sturla
Department of Health Sciences and Technology
ETH Zürich, Zürich, Switzerland, 8092
E-mail: sturlas@ethz.ch
[b] Dr. J. Sun
Department of Biological Engineering
Massachusetts Institute of Technology
77 Massachusetts Avenue, Cambridge, MA 02139, USA
[c] Dr. N. M. Antczak
Department of Chemistry
Skidmore College
815 North Broadway, Saratoga Springs, NY 12866 USA

Experimental details of chemical synthesis	2-7
Table S1	7
Figure S1 – S12	8-14
ESI-MS of molecular beacons	15-16
NMR data	17-24

Reagents and Materials

Solvents and chemical reagents were purchased from Sigma-Aldrich if not specifically mentioned otherwise. Commercial enzymes T4 ligase, Exonuclease I, Exonuclease III, Fpg, APE1, hAAG and UDG were purchased from New England Biolabs and hOGG1 was purchased from R&D system. All reactions were monitored by TLC using commercial Merck Plates coated with silica gel GF254 (0.24 mm thick). Flash column chromatography was performed on a Biotage SP4 system with pre-packed cartridges. ^{1}H and ^{13}C NMR spectra were recorded on a Bruker Biospin 400 MHz NMR instrument operating at 25 °C. Chemical shifts (δ, ppm) were reported relative to the residual solvent peaks, together with coupling constants (*J*). MS data was measured on a Velos Ion Trap Mass spectrometer (Thermo Fisher Scientific).

Synthesis of nitro-phosphoramidite precursor 6

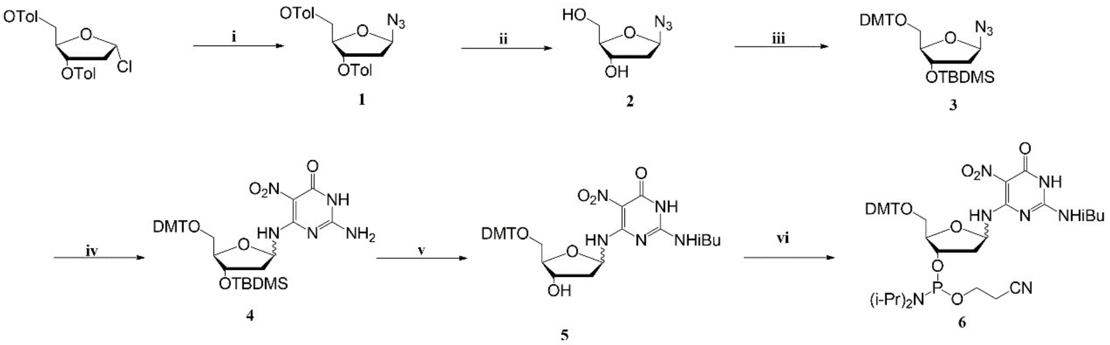

Scheme S1. Synthesis of nitro-phosphoramidite precursor. **i.** Cesium azide, DMSO, yield 68%; **ii.** Sodium hydroxide, methanol, yield 96%; **iii.** a. 4,4-Dimethoxytrityl chloride, pyridine, yield 88%; b. *tert*-Butyldimethylsilyl chloride, imidazole, pyridine, yield 86%; **iv.** a. H_2, Pd/C, methanol; b. 2-Amino-6-chloro-5-nitropyrimidin-4(3H)-one, triethylamine, methanol, yield 51%; **v.** a. Isobutyl chloride, pyridine; b. Tetra-n-butylammonium fluoride, acetonitrile, yield 51% for two steps; **vi.** 2-Cyanoethyl-*N,N*-diisopropylchlorophosphoramidite, triethylamine, dichloromethane.

3,5-Di-O-(p-toluyl)-2-deoxy-D-ribofuranosyl azide (**1**)

3,5-Di-O-(p-toluyl)-2-deoxy-D-ribofuranosyl chloride (3.2 g, 8.3 mmol) was added to a mixture of sodium azide (0.64 g, 9.9 mmol) in 10 mL anhydrous DMSO. The reaction mixture was stirred at room temperature under nitrogen atmosphere and monitored by TLC. After the starting material was consumed, the mixture was poured into ethyl acetate (100 mL), washed with brine (3×100 mL), and dried over Na_2SO_4. The organic layer was concentrated under reduced pressure, and the resulting residue was purified on a silica gel column on a biotage automated chromatography system using isocratic hexane: EtOAc (20:1) as solvent, yielding compound **1** as a single β isomer in the form of white solid (2.2 g, 68%). ^{1}H NMR (400 MHz, Chloroform-*d*) δ 7.94 (dd, *J* = 19.6, 8.0 Hz, 4H), 7.30–7.16 (m, 4H), 5.71 (d, *J* = 6.1 Hz, 1H), 5.49 (dt, *J* = 7.5, 1.9 Hz, 1H), 4.71 (q, *J* = 3.6 Hz, 1H), 4.67–4.46 (m, 2H), 2.54 (dd, *J* = 14.3, 7.1 Hz, 1H), 2.42 (d, *J* = 3.8 Hz, 6H), 2.23 (d, *J* = 14.7 Hz, 1H). ^{13}C NMR (101 MHz, $CDCl_3$) δ 166.31, 166.16, 144.26, 144.03, 129.86, 129.69, 129.23, 126.88, 126.70, 92.05, 83.59, 74.58, 64.00, 38.88, 21.73, 21.70. LTQ-MS: Calc.: 395.15, Found: $[M-N_3]^+$ 353.20

2-deoxy-D-ribofuranosyl azide (**2**)

Compound **1** (2.2 g, 5.6 mmol) was dissolved in 20 mL MeOH and cooled in an ice bath. Sodium methoxide (2.4 g, 45 mmol) in 4 mL MeOH was added over the course of 5 min. The reaction mixture was stirred at room temperature and the progress of the reaction was monitored by TLC. Once compound **1** was totally consumed, the reaction mixture was concentrated to dryness under reduced pressure. The residue was purified on a silica gel column on a biotage system using a gradient of DCM: MeOH (95:5-90:10) as solvent, yielding compound **2** as a mixture of anomers in the form of white solid (0.86 g, 96%). ^{1}H NMR (400 MHz, Methanol-d_4) δ 5.56 (dd, *J* = 5.7, 4.0 Hz, 1H), 4.27 (q, *J* = 5.6 Hz, 1H), 3.90 (q, *J* = 5.1 Hz, 1H), 3.62 (qd, *J* = 11.7, 5.5 Hz, 2H), 2.17–1.99 (m, 2H). ^{13}C NMR (101 MHz, MeOD) δ 93.25, 88.70, 72.35, 64.00, 41.71. LTQ-MS: Calc.: 159.06, Found: $[M+CH_3COO]^-$ 218.34

3-O-*tert*-butyldimethylsilyl-5-O-(4,4′-Dimethoxytrityl)-2-deoxy-D-ribofuranosyl azide (**3**)

Compound **2** (0.52 g, 3.3 mmol) was dissolved in 5 mL anhydrous pyridine, and the reaction mixture was cooled in an ice bath. A solution of 4,4′-dimethoxytrityl chloride (1.3 g, 3.9 mmol) in 5 mL pyridine was added through a syringe under nitrogen atmosphere and the reaction mixture stirred at room temperature for 3 h. After completion of the DMT-azidofuran, imidazole (0.89 g, 13 mmol) and TBDMSCl (0.99 g, 6.5 mmol) were added to the reaction mixture. The reaction mixture was stirred at room temperature overnight and was evaporated to remove solvent. The residue was dissolved in DCM and washed with saturated $CuSO_4$ solution followed by saturated $NaHCO_3$ and brine, and dried over Na_2SO_4. The organic phase was evaporated, and the residue was purified by silica gel column on a biotage system using a gradient of EtOAc: hexanes (15:85-30:70) as solvent, yielding compound **3** (1.7 g, 87%) as a single β isomer in the form of colorless oil. ^{1}H NMR (400 MHz, Chloroform-*d*) δ 7.52 (dd, *J* = 7.9, 2.7 Hz, 2H), 7.44-7.36 (m, 4H), 7.33-7.28 (m, 2H), 7.25-7.19 (m, 1H), 6.89-6.80 (m, 4H), 5.67-5.48 (m, 1H), 4.37 (dd, *J* = 5.5, 2.3 Hz, 1H), 3.99 (s, 1H), 3.86-3.76 (m, 6H), 3.30-3.13 (m, 2H), 2.02 (ddd, *J* = 9.4, 6.3, 2.7 Hz, 2H), 0.82 (d, *J* = 2.5 Hz, 9H), -0.03 (dd, *J* = 25.2, 2.5 Hz, 6H). ^{13}C NMR (101 MHz, $CDCl_3$) δ 158.46, 144.82, 136.08, 130.09, 128.23, 127.81, 126.72, 113.12, 91.72, 86.18, 86.11, 71.87, 63.65, 55.21, 41.11, 25.67, 17.88, -4.76, -4.94. LTQ-MS: Calc.: 575.28, Found: $[M+Na]^+$ 598.25

Compound **4**

Compound **3** (0.49 g, 0.85 mmol) was dissolved in 5 mL anhydrous ethanol and Pd on carbon (5 %, 0.16 g) was added as a catalyst. The mixture exposed to an atmosphere of H_2 until starting material was consumed. The resulting mixture was filtered quickly through celite and triethylamine (0.24 mL, 1.7 mmol) was added to the filtrate**,** followed by 2-amino-4-chloro-5-nitro-6-pyrimidinone (0.24 g, 1.3 mmol). The reaction mixture was heated under reflux overnight under a nitrogen atmosphere, cooled, and evaporated under reduced pressure to yield a solid residue. The solid was purified by silica gel column on a biotage system using isocratic DCM: MeOH (95:5) as solvent, yielding compound **4** (0.30 g, 51%) as mixture of anomers in a form of yellow powder. ^{1}H NMR (400 MHz, Chloroform-d) δ 10.70 (s, 0.5H), 10.18-9.70 (m, 1H), 8.32 (d, J = 32.4 Hz, 0.5H), 7.37-7.12 (m, 9H), 6.79-6.65 (m, 4H), 6.36-5.86 (m, 1H), 5.63-5.18 (m, 1H), 4.31-4.26 (m, 1H), 4.15-3.86 (m, 1H), 3.68-3.66 (m, 6H), 3.16-2.93 (m, 2H), 2.35-2.13 (m,

1H), 1.93-1.78 (m, 1H), 1.19-1.09 (m, 1H), 0.80-0.74 (m, 9H), 0.00-0.11 (m, 6H). ^{13}C NMR (101 MHz, $CDCl_3$) δ 158.91, 158.51, 153.99, 144.77, 135.93, 130.09, 130.06, 130.00, 128.14, 127.98, 127.84, 126.82, 113.36, 113.16, 111.31, 87.40, 86.43, 86.31, 81.99, 74.52, 73.06, 63.72, 55.25, 55.21, 40.22, 26.01, 25.79, 18.39, 18.02, -4.57, -4.66. LTQ-MS: Calc.: 703.30, Found: $[M+H]^+$ 704.39

Compound **5**

Isobutyryl chloride (0.026 g, 0.24 mmol) was added to a solution of compound **4** (0.14 g, 0.20 mmol) in 5 mL dry pyridine. The mixture was stirred at room temperature for 2 h. Pyridine was evaporated under vacuum and the resulting residue was dissolved in DCM (100 mL), washed with brine, and dried over Na_2SO_4. The organic layer was concentrated under reduced pressure, and the resulting residue was dissolved in THF (2 mL). TBAF·$3H_2O$ (0.13 g, 0.41 mmol) was added and the reaction mixture was stirred at room temperature for 4h. 50 mL of DCM was added to the resultant mixture and washed with brine (3×50 mL), dried over Na_2SO_4 and the solvent was evaporated under reduced pressure. The residue was purified by silica gel column on a biotage system using isocratic DCM: MeOH (97:3) as solvent, yielding compound **5** (0.10 g, 75%) as a mixture of anomers in a form of colorless foam. ^{1}H NMR (400 MHz, Chloroform-d) δ 11.44 (s, 0.6H), 10.39-9.51 (m, 1H), 8.73 (s, 0.4H), 7.32-7.06 (m, 9H), 6.74-6.68 (m, 4H), 6.12-5.96 (m, 1H), 4.39-4.24 (m, 1H), 4.19-3.91 (m, 1H), 3.70-3.64 (m, 6H), 3.12-2.95 (m, 2H), 2.62-2.39 (m, 1H), 2.32-2.21 (m, 1H), 2.17-1.85 (m, 2H), 1.10-1.08 (m, 6H). ^{13}C NMR (101 MHz, $CDCl_3$) δ 180.21, 179.30, 158.96, 158.55, 158.53, 158.02, 154.98, 154.89, 150.75, 150.21, 144.77, 144.53, 135.87, 135.78, 135.60, 130.08, 130.05, 128.10, 128.06, 127.92, 127.88, 126.91, 113.61, 113.32, 113.23, 113.22, 113.20, 86.62, 86.50, 86.38, 85.83, 82.29, 82.07, 73.48, 73.12, 64.22, 63.93, 55.28, 55.24, 40.46, 39.74, 36.52, 36.32, 18.77, 18.69, 18.63. LTQ-MS: Calc.: 659.26, Found: $[M-H]^-$ 658.23

Compound **6**

TEA (14 µL, 0.10 mmol) and 2-Cyanoethyl-*N,N*-diisopropylchlorophosphoramidite (18 mg, 0.075 mmol) was added to a solution of compound **5** (33 mg, 0.050 mmol) in 2 mL anhydrous DCM. After 3 h the reaction mixture was poured into 20 mL DCM, washed with cold saturated $NaHCO_3$, dried over Na_2SO_4 and concentrated under vacuum. The resulting residue was dissolved in 300 µL anhydrous CH_3CN, and molecular sieves were added for 1 h. The solution was used for standard DNA solid phase synthesis without further purification. LTQ-MS: Calc.: 859.37, Found: $[M-H]^-$ 858.48

Synthesis of 5′-amino-deoxyguanosine phosphoramidite

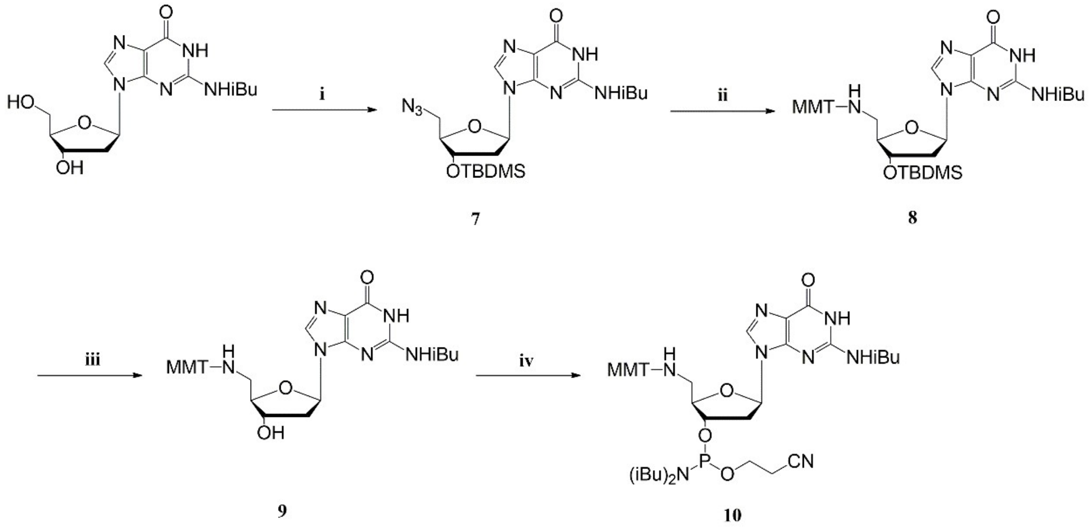

Scheme S2. Synthesis of 5′ amino guanosine phosphoramidate. **i.** a. *p*-Toluenesulfonyl chloride, pyridine; b. *tert*-Butyldimethylsilyl chloride, imidazole; c. Sodium azide, DMF; yield 76%; **ii.** a. 10% Palladium on carbon, EtOH; b. 4-Monomethoxytrityl chloride, pyridine; yield 68%; **iii.** Tetra-n-butylammonium fluoride, acetonitrile; yield 84%; **iv.** 2-Cyanoethyl-*N*,*N*-diisopropylchlorophosphoramidite, triethylamine, dichloromethane.

N^2-isobutyryl-3′-O-*tert*-butyldimethylsilyl-5′-azido-2′, 5′-dideoxyguanosine (**7**)

N^2-isobutyryl-2′, 5′-dideoxyguanosine (1.0 g, 3.0 mmol) was dissolved in 10 mL anhydrous pyridine. A solution of 4-toluenesulfonyl chloride (0.63 g, 3.3 mmol) in 5 mL pyridine was added through a syringe under nitrogen atmosphere and the reaction mixture was stirred at room temperature for 6 h. Then, Imidazole (0.41 g, 6.0 mmol) and TBDMSCl (0.91 g, 6.0 mmol) were added to the reaction mixture. The reaction mixture was stirred at room temperature overnight and was evaporated to remove solvent. The residue was dissolved in 100 mL DCM and washed with saturated $CuSO_4$ solution (100 mL) followed by $NaHCO_3$ (100 mL) and brine (100 mL), and dried over Na_2SO_4. The organic phase was evaporated, and the residue was dissolved in 10 mL DMF. Sodium azide (0.65 g, 10 mmol) was added and the reaction mixture was stirred at 50 °C for 6 h. The reaction mixture was poured into DCM (100 mL), washed with brine (3×50 mL), dried over Na_2SO_4 and the solvent was evaporated under reduced pressure. The residue was purified by silica gel column on a biotage system using isocratic DCM: MeOH (95:5) as solvent, yielding compound **7** (0.83 g, 76%) as a white solid. ^{1}H NMR (400 MHz, Chloroform-*d*) δ 12.17 (s, 1H), 9.27 (s, 1H), 7.94 (s, 1H), 6.20 (t, *J* = 6.5 Hz, 1H), 4.49 (dt, *J* = 6.0, 4.2 Hz, 1H), 4.01 (q, *J* = 4.3 Hz, 1H), 3.54 (qd, *J* = 13.2, 4.5 Hz, 2H), 2.81-2.67 (m, 2H), 2.38-2.34 (m, 1H), 1.25 (dd, *J* = 6.9, 5.1 Hz, 6H), 0.89 (s, 9H), 0.10 (s, 6H). ^{13}C NMR (101 MHz, $CDCl_3$) δ 179.01, 155.68, 148.27, 147.89, 137.34, 121.49, 85.71, 84.09, 72.40, 51.95, 40.35, 36.54, 25.81, 19.11, 18.06, -4.57, -4.73. LTQ-MS: Calc.: 476.23, Found: $[M+H]^+$ 477.16

N^2-isobutyryl-3′-O-*tert*-butyldimethylsilyl-5′-(4-methoxytrityl) amino-2′,5′-dideoxyguanosine (**8**)

Pd on carbon (10 %, 1.2 g) was added to a solution of compound **7** (0.80 g, 2.2 mmol) in 20 mL ethanol. Hydrogen gas was bubbled through the reaction and the mixture was stirred vigorously at room temperature for 6 h. The solution was filtered through Celite, washed with

around 10 mL of ethanol, then concentrated under reduced pressure. The residue was co-evaporated twice with anhydrous pyridine (10 mL) and dissolved in 5 mL anhydrous pyridine. 4-Methoxytrityl chloride (1.5 g, 5.0 mmol) was added to the reaction mixture. The reaction mixture was stirred at room temperature overnight and then evaporated to remove solvent. The residue was dissolved in 50 mL DCM and washed with saturated $CuSO_4$ solution (50 mL) followed by $NaHCO_3$ (50 mL) and brine (50 mL) and dried over Na_2SO_4. The solvent was evaporated under reduced pressure and the residue was purified by silica gel column on a biotage system using isocratic DCM: MeOH (97:3) as solvent, yielding compound **8** (1.1 g, 68%) as a yellow foam. ^{1}H NMR (400 MHz, Chloroform-*d*) δ 11.97 (s, 1H), 7.90 (s, 1H), 7.57 (s, 1H), 7.46-7.39 (m, 4H), 7.35-7.31 (m, 2H), 7.26-7.23 (m, 4H), 7.20-7.15 (m, 2H), 6.78 (d, *J* = 8.9 Hz, 2H), 6.14 (dd, *J* = 7.7, 5.8 Hz, 1H), 4.44 (dt, *J* = 6.1, 3.2 Hz, 1H), 4.06 (dt, *J* = 6.3, 3.2 Hz, 1H), 3.75 (s, 3H), 2.60-2.55 (m, 2H), 2.24-2.19 (m, 1H), 2.05-2.01 (m, 1H), 1.87-1.81 (m, 2H), 1.08 (d, *J* = 6.9 Hz, 3H), 1.04 (d, *J* = 6.9 Hz, 3H), 0.85 (s, 9H), 0.01 (d, *J* = 9.2 Hz, 6H). ^{13}C NMR (101 MHz, $CDCl_3$) δ 178.29, 158.27, 155.51, 147.94, 147.33, 146.04, 137.89, 137.29, 129.90, 128.63, 128.16, 126.73, 122.43, 113.44, 87.90, 84.37, 73.64, 70.18, 68.11, 55.37, 45.91, 41.26, 36.54, 25.88, 25.75, 19.00, 18.11, -4.53, -4.62. LTQ-MS: Calc.: 722.36, Found: $[M+H]^+$ 723.30

N^2-isobutyryl-5′-(4-methoxytrityl)amino-2′,5′-dideoxyguanosine (**9**)

TBAF·$3H_2O$ (0.88 g, 2.8 mmol) was added to a solution of compound **8** (1.0 g, 1.4 mmol) in 10 mL THF. The mixture was stirred at room temperature for 6 h. THF was evaporated under vacuum and the residue was purified by silica gel column on a biotage system using isocratic DCM: MeOH (95:5) as solvent, yielding compound **9** (0.72 g, 84%) as a white solid. ^{1}H NMR (400 MHz, Chloroform-*d*) δ 7.61 (s, 1H), 7.38 (dd, *J* = 8.4, 1.3 Hz, 4H), 7.29 (d, *J* = 8.8 Hz, 2H), 7.21 (dd, *J* = 8.3, 6.7 Hz, 4H), 7.16-7.10 (m, 2H), 6.75 (d, *J* = 8.9 Hz, 2H), 6.17 (t, *J* = 6.5 Hz, 1H), 4.74 (d, *J* = 5.5 Hz, 1H), 4.14 (dt, *J* = 7.9, 4.0 Hz, 1H), 3.74 (s, 3H), 2.67-2.58 (m, 2H), 2.48-2.40 (m, 1H), 2.34-2.28 (m, 2H), 1.14 (dd, *J* = 10.4, 6.8 Hz, 6H). ^{13}C NMR (101 MHz, $CDCl_3$) δ 179.15, 158.19, 155.68, 148.11, 147.65, 146.02, 137.85, 137.71, 129.92, 128.64, 128.06, 126.64, 121.93, 113.37, 87.18, 84.20, 72.78, 70.24, 55.37, 46.15, 40.50, 36.46, 19.09, 19.02. LTQ-MS: Calc.: 608.28, Found: $[M+H]^+$ 608.89

N^2-isobutyryl-5′-(4-methoxytrityl) amino-2′,5′-dideoxyguanosine, 3′-[(2-cyanoethyl)-(*N,N*-diisopropyl)]-phosphoramidite (**10**)

TEA (28 µL, 0.20 mmol) and 2-Cyanoethyl-*N,N*-diisopropylchlorophosphoramidite (27 mg, 0.15 mmol) were added to a solution of compound **9** (61 mg, 0.10 mmol) in 2 mL anhydrous DCM. After 3 h the reaction mixture was poured into 20 mL DCM, washed with cold saturated $NaHCO_3$, dried over Na_2SO_4 and evaporated under vacuum. Then the residue was re-dissolved in 300 µL anhydrous CH_3CN and dried with molecular sieves for 1 h. The phosphoramidite was used for standard DNA solid phase synthesis without further purification. LTQ-MS: Calc.: 808.38, Found: $[M+H]^+$ 808.80

Table S1. Summary of molecular beacon characteristics and mass spectrometry data.

	5′	3′	Sequence	Calculated M.W.	Found M.W.
MB-FAM-G	FAM	BHQ1	**AM-dG**GT CTG ATG GGA GGC GAC ACC TCC CAT CAG ACC	11030	11028
MB-Cy3-FapyG	Cy3	BHQ2	**AM-dG**GT CT**FapyG** ATG GGA GGC GAC ACC TCC CAT CAG ACC	11130	11130
MB-Cy5-8oxoG	Cy5	BHQ2	**AM-dG**GT CT**8-oxoG** ATG GGA GGC GAC ACC TCC CAT CAG ACC	11154	11154

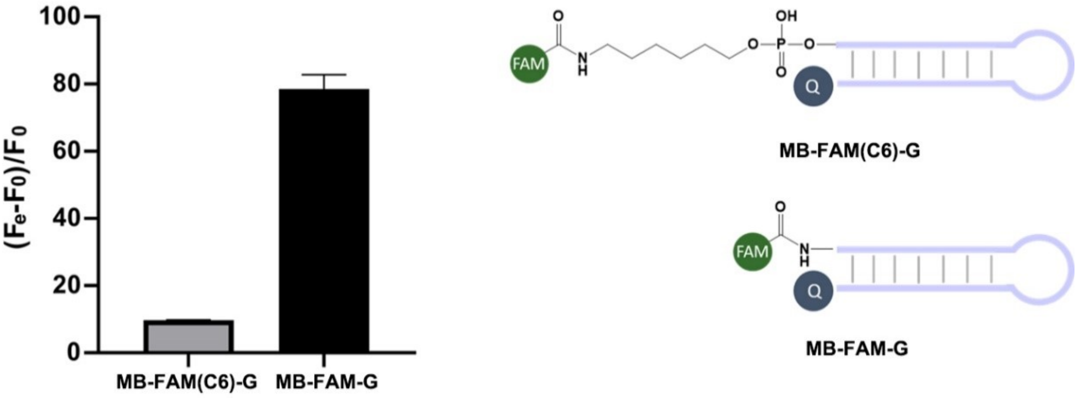

Figure S1. Signal-to-noise ratio of fluorescence measurement of molecular beacons containing a C6 amino modifier (MB-FAM(C6)-G) vs. amino guanosine (MB-FAM-G). F_0 is the fluorescence signal of probes in 1× cutsmart buffer (0.5 µM probes, 50 mM potassium acetate, 20 mM tris-acetate, 10 mM magnesium acetate, 100 µg/ml BSA, pH 7.9). F_e is the fluorescence signal of the same probes in presence of Exo **I** & Exo **III** (0.01 U Exo **I**, 0.1 U Exo **III**) in the same buffer as F_0.

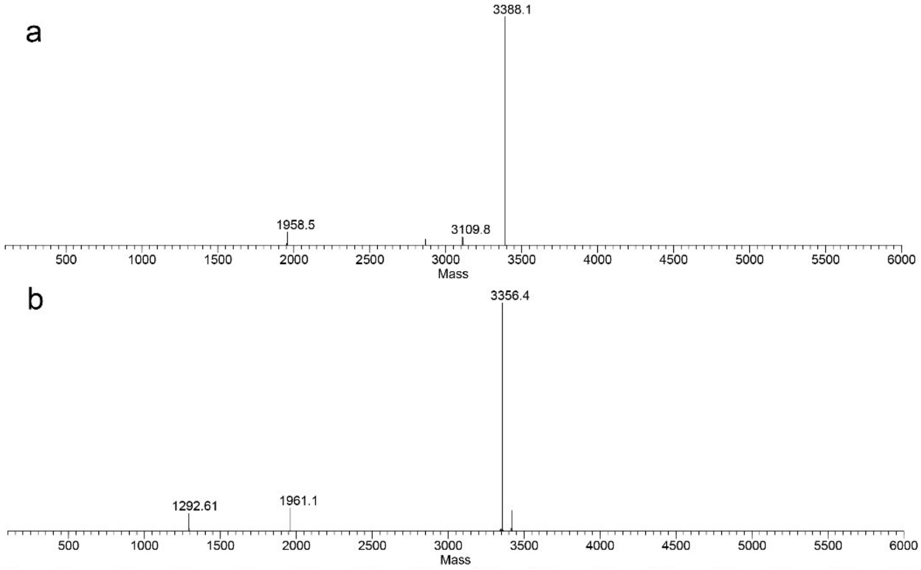

Figure S2. (a) Mass spectrum of oligonucleotide containing NP-dG (Scheme 1, **II**). (b) Mass spectrum of oligonucleotide containing FapyG (Scheme 1, **III**) which was reduced from NP-dG by $NaBH_4/Ni_2B$.

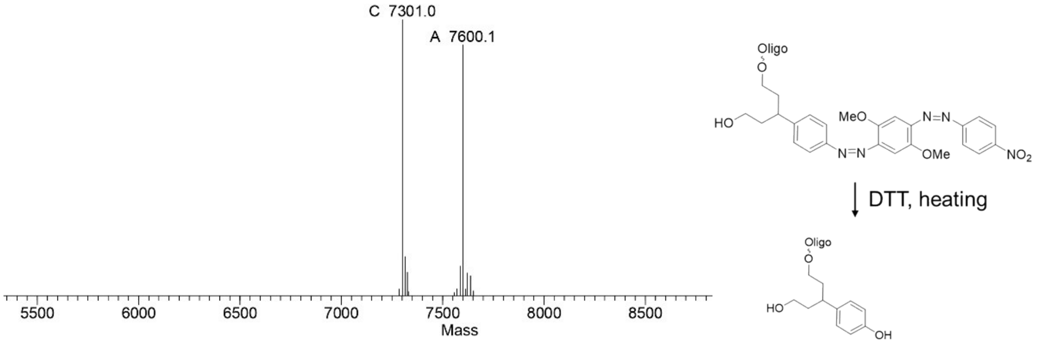

Figure S3. Evidence for the cleavage of the BHQ2 moiety in the presence of DTT when BHQ2-containing oligonucleotides (M.W. 7600) were allowed to react with T4 DNA ligase (2000U, 50 mM Tris-HCl, 10 mM $MgCl_2$, 1 mM ATP, 10 mM DTT) at 95 °C for 10 min. The cleavage product had M.W. 7301.

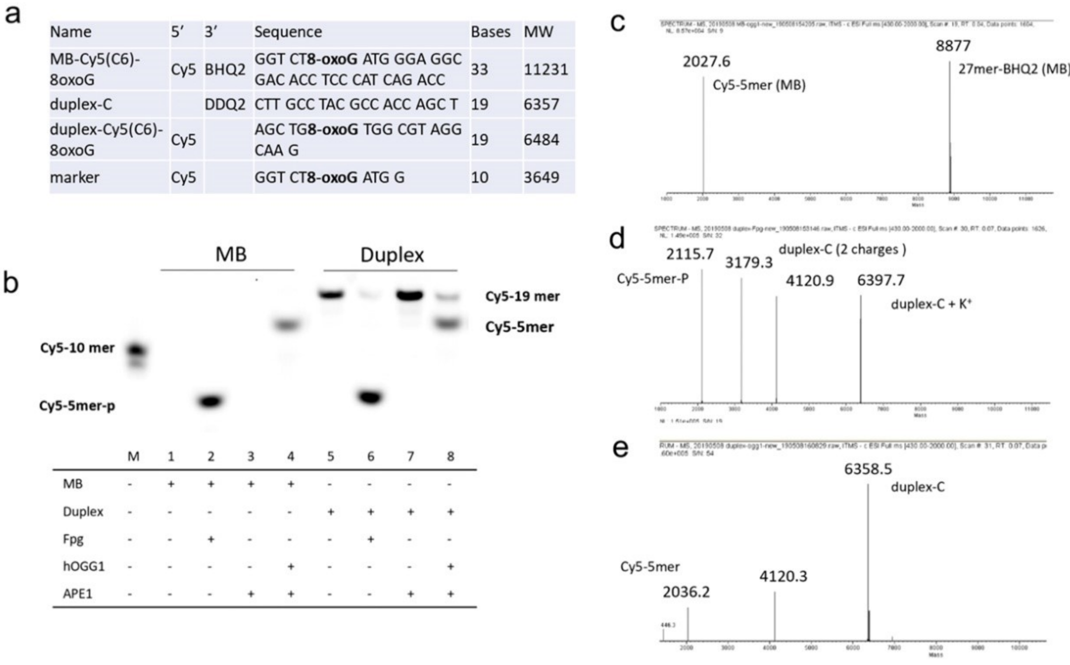

Name	5′	3′	Sequence	Bases	MW
MB-Cy5(C6)-8oxoG	Cy5	BHQ2	GGT CT**8-oxoG** ATG GGA GGC GAC ACC TCC CAT CAG ACC	33	11231
duplex-C		DDQ2	CTT GCC TAC GCC ACC AGC T	19	6357
duplex-Cy5(C6)-8oxoG	Cy5		AGC TG**8-oxoG** TGG CGT AGG CAA G	19	6484
marker	Cy5		GGT CT**8-oxoG** ATG G	10	3649

	M	1	2	3	4	5	6	7	8
MB	-	+	+	+	+	-	-	-	-
Duplex	-	-	-	-	-	+	+	+	+
Fpg	-	-	+	-	-	-	+	-	-
hOGG1	-	-	-	-	+	-	-	-	+
APE1	-	-	-	+	+	-	-	+	+

Figure S4. PAGE gel and mass spectrometric analysis of glycosylase-mediated cleavage of probes. (a) List of molecular beacon and duplex sequences (duplex-C and duplex-Cy5(C6)-8oxoG are complementary sequences and were annealed before use). (b) PAGE gel analysis of reactions of MBs (Lane 1 to 4) and duplexes (Lane 5 to 8) with Fpg, hOGG1 or hOGG1/APE1. (c) Mass spectrum of lane 4 in (b); (d) Mass spectrum of lane 6 in (b); (e) Mass spectrum of lane 8 in (b).

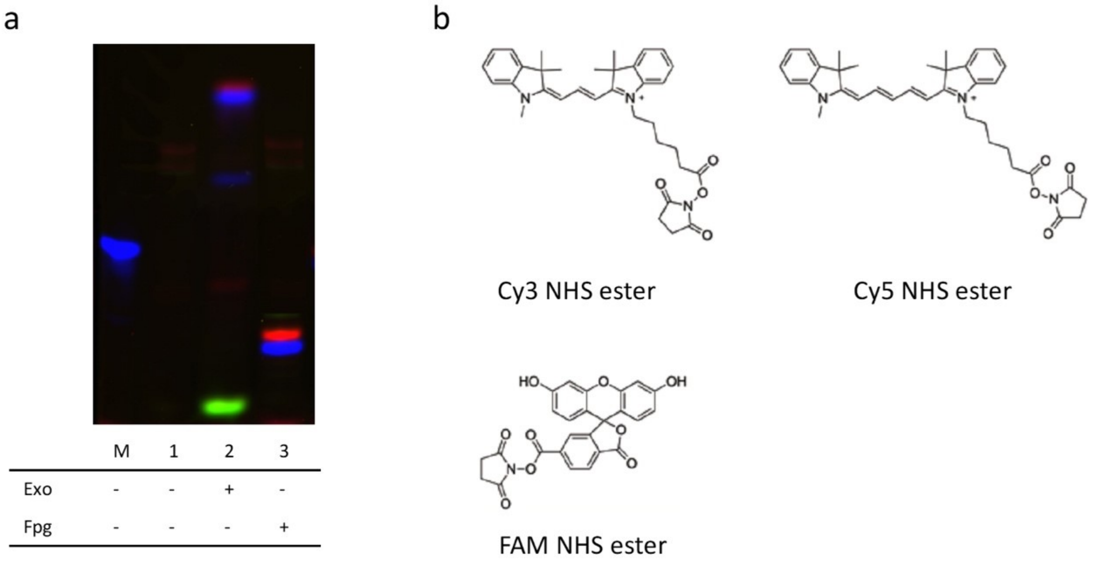

Figure S5. (a) PAGE gel analysis of exonuclease- & Fpg-mediated molecular beacon cleavage. Lane M indicates a Cy3-labeled 5mer marker. The green, blue and red bands indicate the cleavage products of MB-FAM-G, MB-Cy3-FapyG and MB-Cy5-8oxoG. The gel migration of Cy3/Cy3-oligonucleotide is faster than Cy5/Cy5-oligonucleotide (exonuclease cleavage products in Lane 1 and Fpg cleavage products in Lane 3). Similar migration difference has been observed for Cy3/Cy5 labelled GTP.[1] Cy3/Cy5 have different stacking interactions between indole rings of dyes and terminal bases induced by their different polymethine linker lengths. This interaction difference may exert an impact on the gel shift of Cy3/Cy5-oligonucleotide.[2] (b) Chemical structures of NHS esters of the dyes used in this study.

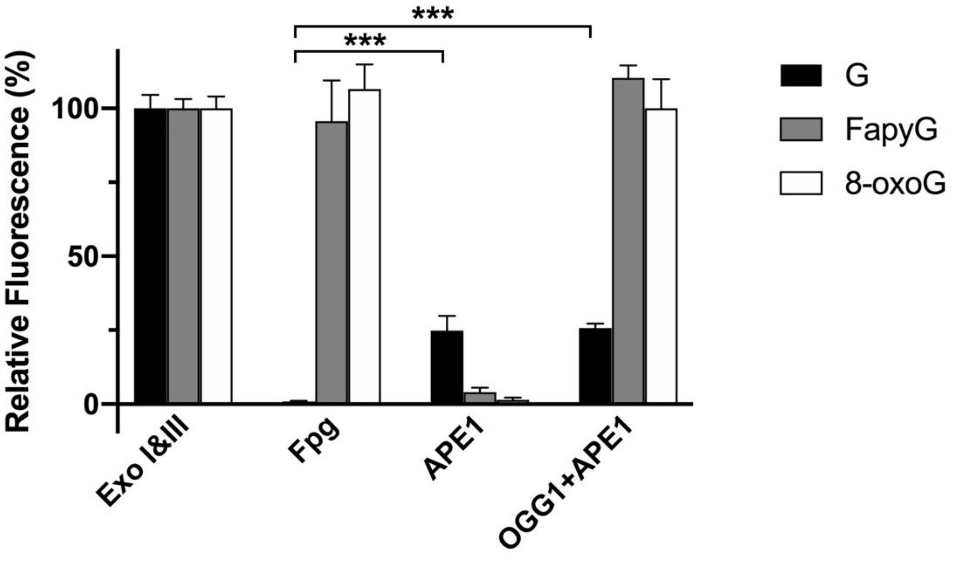

Figure S6. Cleavage of molecular beacons in the presence of different enzymes with Mg^{2+} added to 1×cutsmart buffer (50 mM potassium acetate, 20 mM tris-acetate, 10 mM magnesium acetate, 100 µg/ml BSA, pH 7.9) with Exonuclease I (0.01 U/µL), Exonuclease III (0.1 U/µL),

hOGG1 (0.5 μM), Fpg (0.25 μM) and APE1 (0.25 μM). *P* values were determined by one-way ANOVA. *** indicates $P<.001$, n=4.

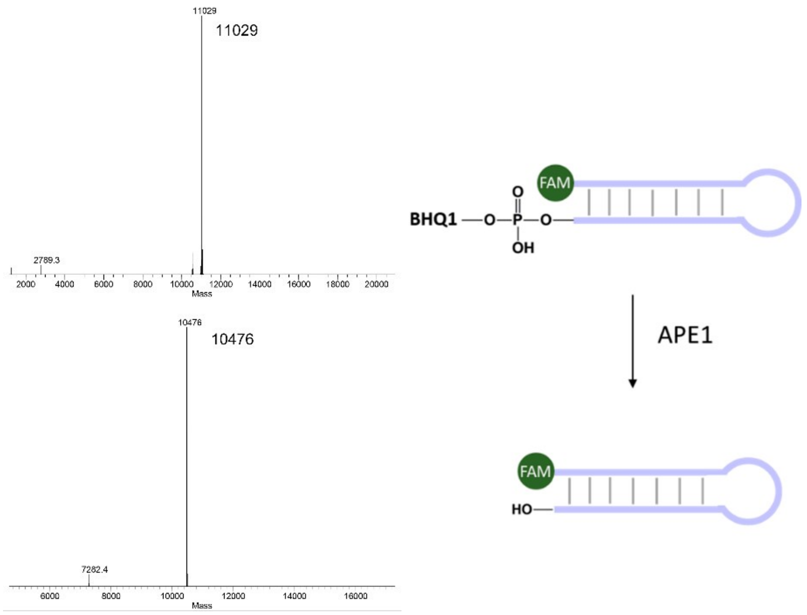

Figure S7. Cleavage of a BHQ1-containing molecular beacon by APE1. After cleavage by APE1, the quencher-free oligonucleotide was purified by PAGE and its identity confirmed by MS.

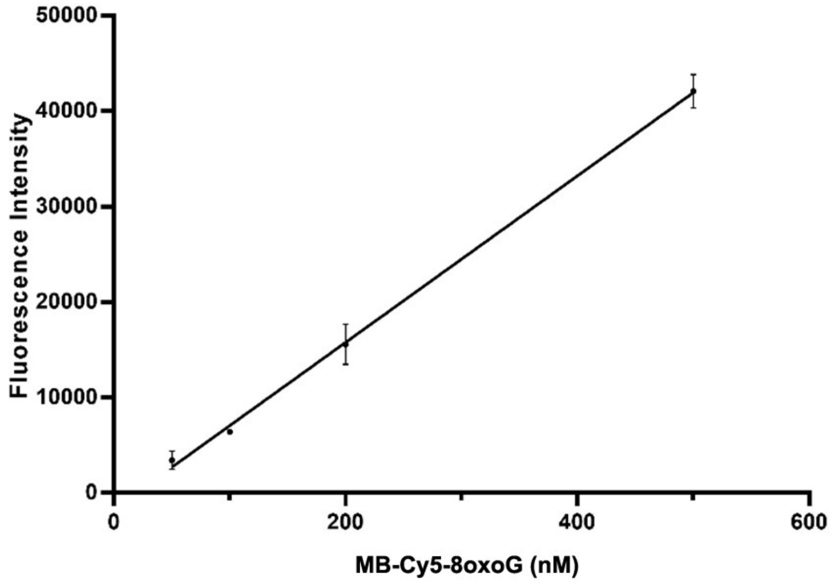

Figure S8. Linear relationship between the Cy5 signal and the concentration of MB-Cy5-8oxoG treated with hOGG1 and DMEDA, ranging from 50 nM to 500 nM.

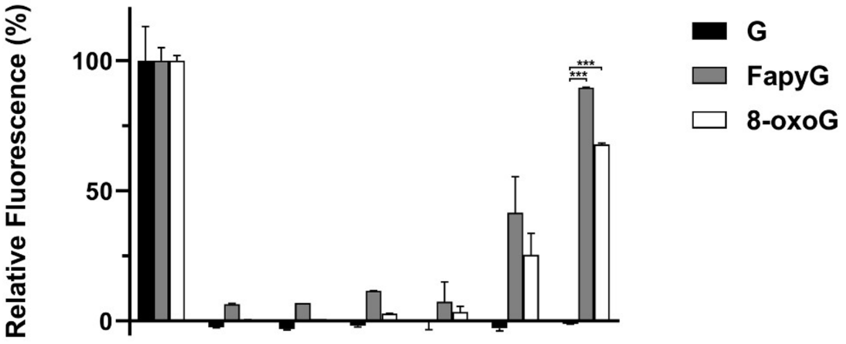

Number of BL21 cells (EV)	10^6	10^4	10^5	10^6	-	-	-
Number of BL21 cells (hOGG1)	-	-	-	-	10^4	10^5	10^6
Mg^{2+} (10 mM)	+	-	-	-	-	-	-

Figure S9. Relative fluorescence intensity of molecular beacons in the presence of cell lysates from different numbers of *E.coli* cells (0.5 μM probes, 50 mM potassium Acetate, 20 mM tris-acetate, 1 mM EDTA, 100 μg/mL BSA, pH 7.9). EV refers to empty vector transformed into *E. coli* BL21 cells; hOGG1 refers to hOGG1 plasmid transformed into *E. coli* BL21 cells. The number in the table refers to the cell amount applied in the assays. *P* values were determined by one-way ANOVA. *** indicates *P*<.001, n=6.

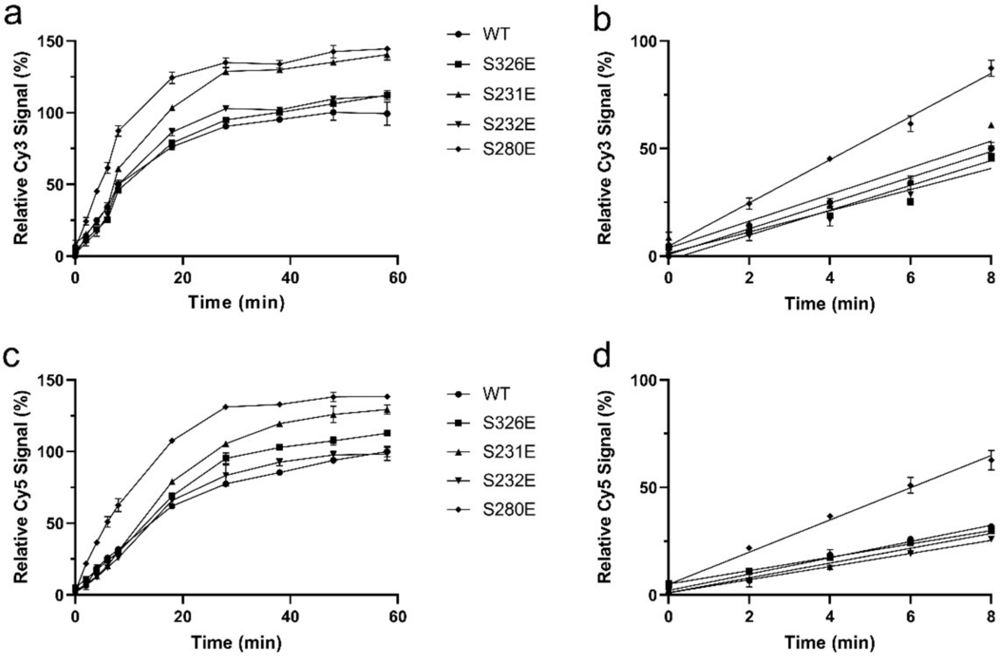

Figure S10. Kinetics for enzymatic excision by hOGG1 phosphorylation mimics (S326E, S231E, S232E and S280E). The signals from WT hOGG1 after 1 h incubation was set to 100%. The signals from WT hOGG1 after 1 h incubation was set to 100%. The Cy3 (FapyG) (b) and Cy5 (8-oxoG) (d) signals in the first 8 min were used for linear regression to determine relative initial reaction rates, n=4.

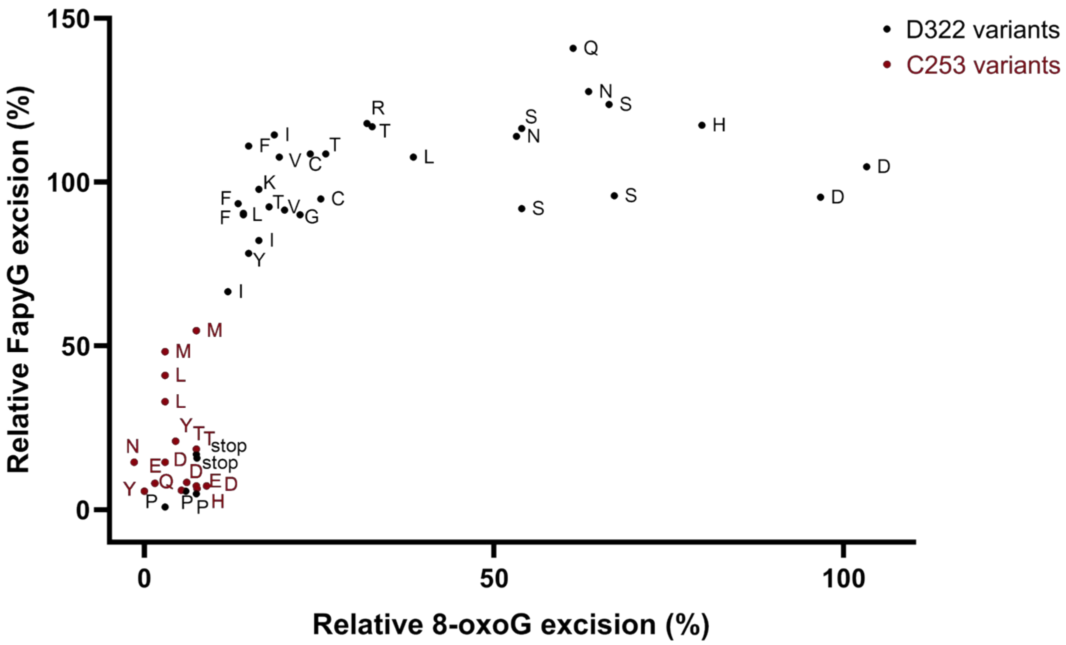

Figure S11. Relative activity of hOGG1 D322 (black) and C253 (red) variants on FapyG (Cy3 signal) and 8-oxoG (Cy5 signal). Average activity of two wild type (D in black) single colonies was set to 100%. Each dot indicates the result for an individually selected variant colony, labeled with the single-letter amino acid code for the amino acid that was present in the mutant variant.

References

1. L. Büttner, F. Javadi-Zarnaghi and C. Höbartner, *Journal of the American Chemical Society*, 2014, **136**, 8131-8137.
2. O. Kroutil, I. Romancova, M. Sip and Z. Chval, *J Phys Chem B*, 2014, **118**, 13564-13572.

ESI-MS data for oligonucleotides and molecular beacons

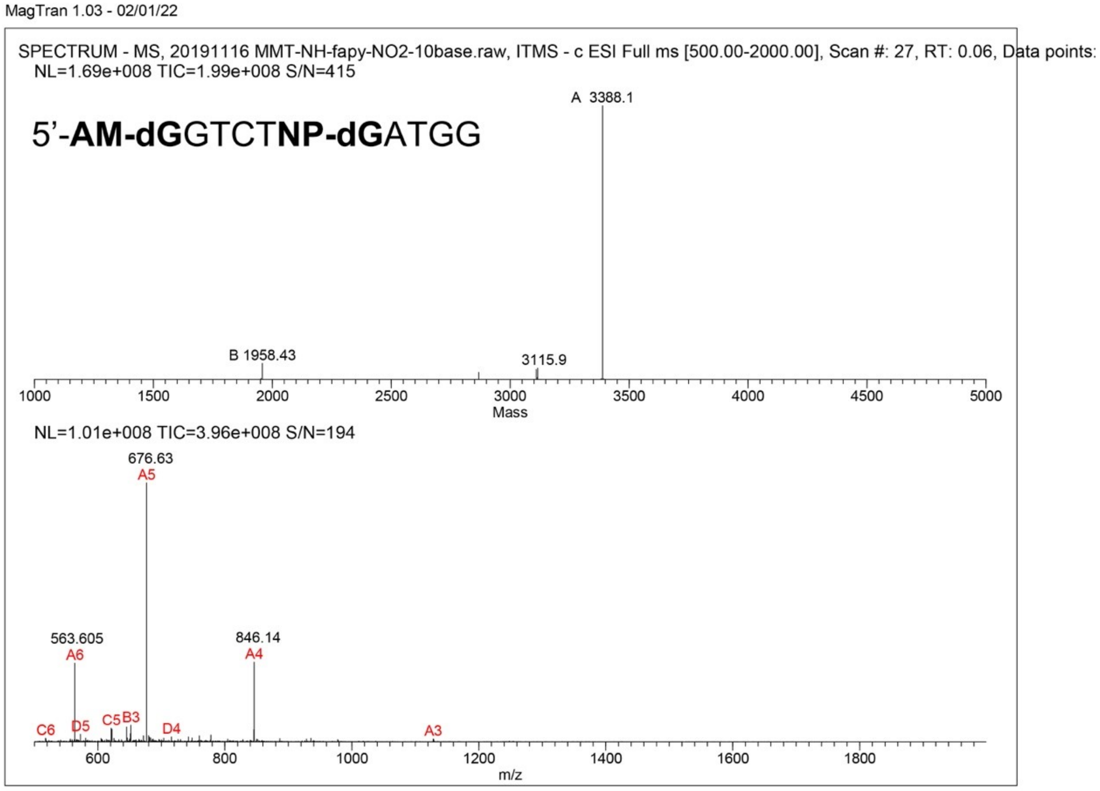

MB-FAM-G

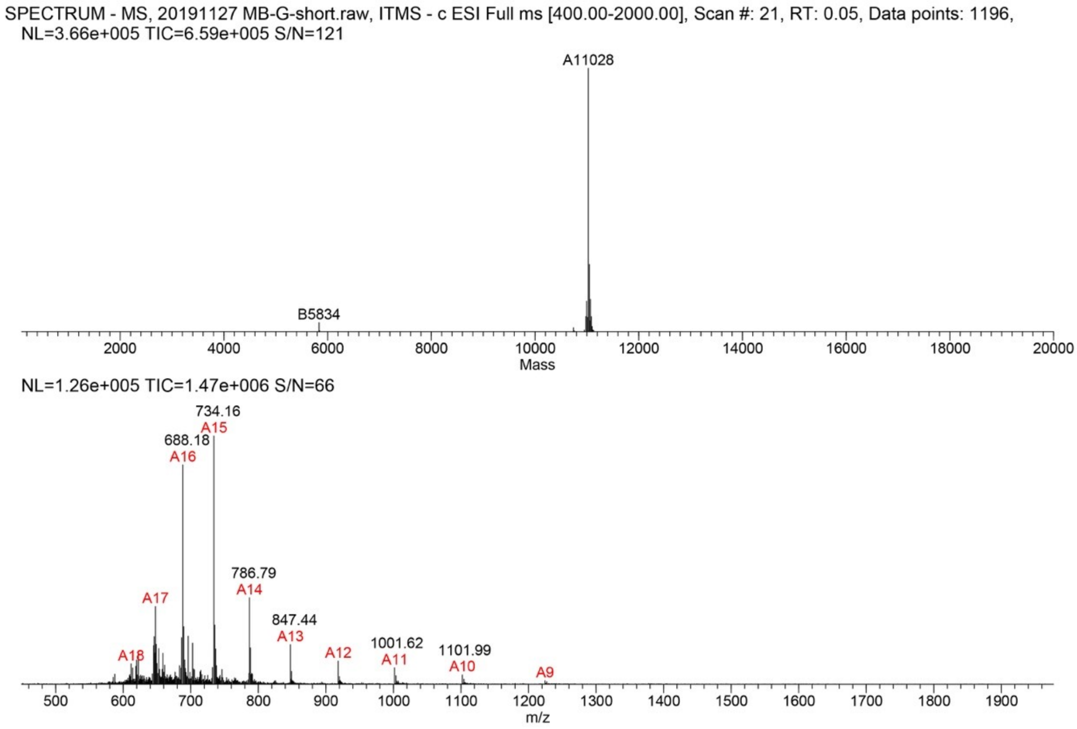

MB-Cy5-8oxoG

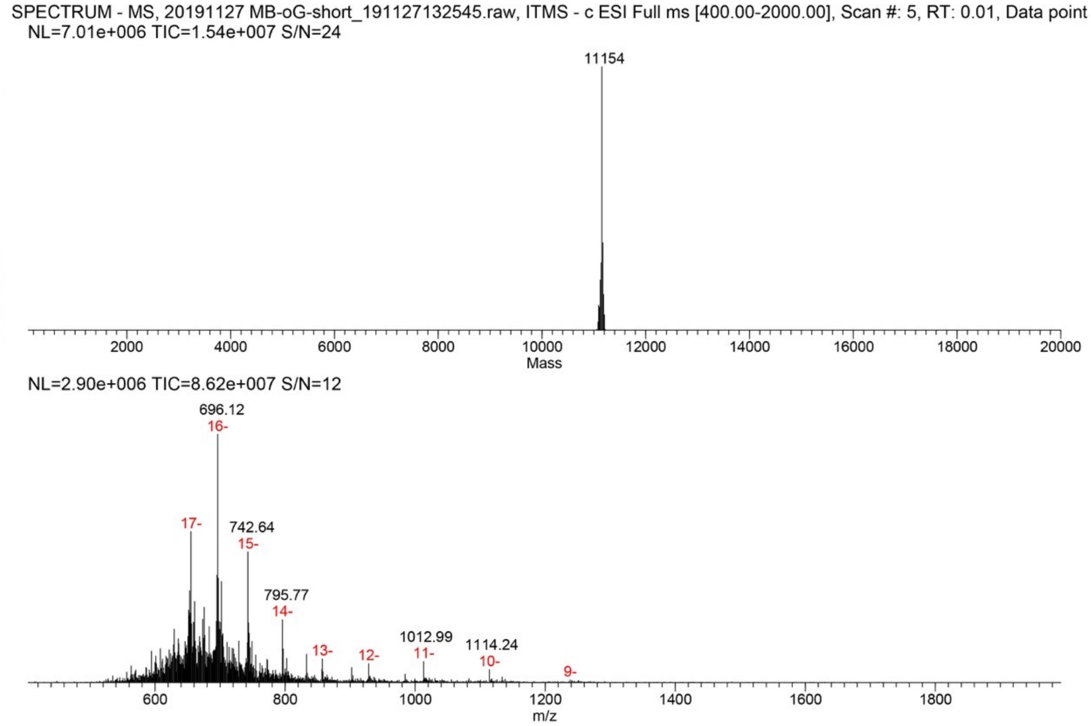

MB-Cy3-FapyG

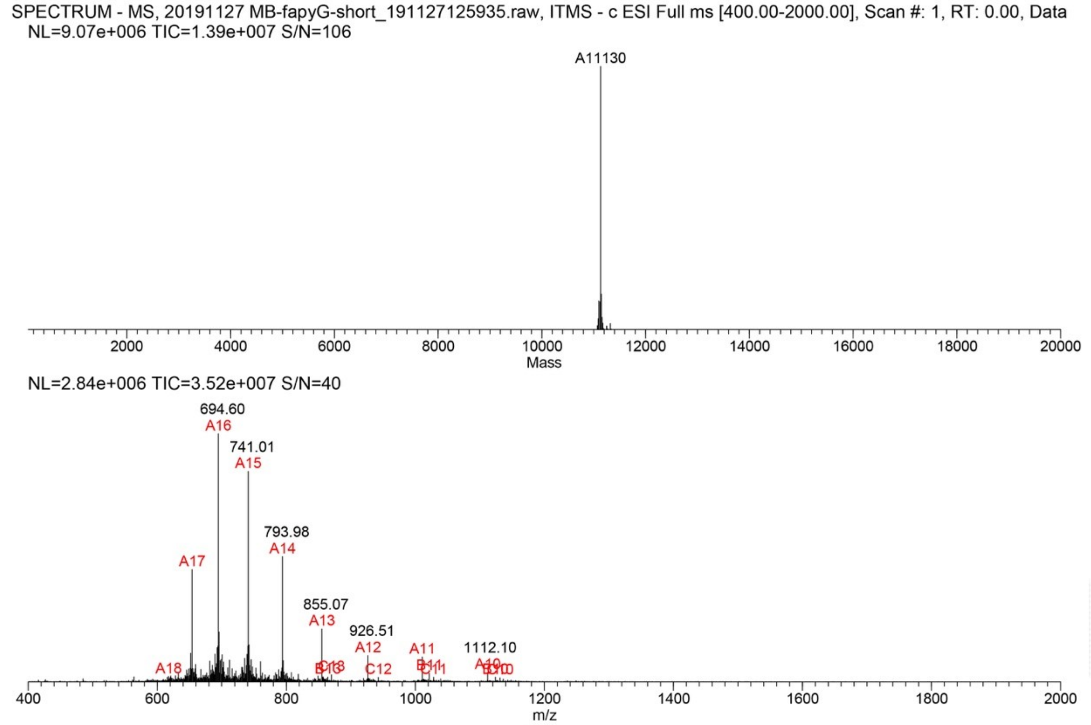

NMR (^1H and ^{13}C) data

Compound 1

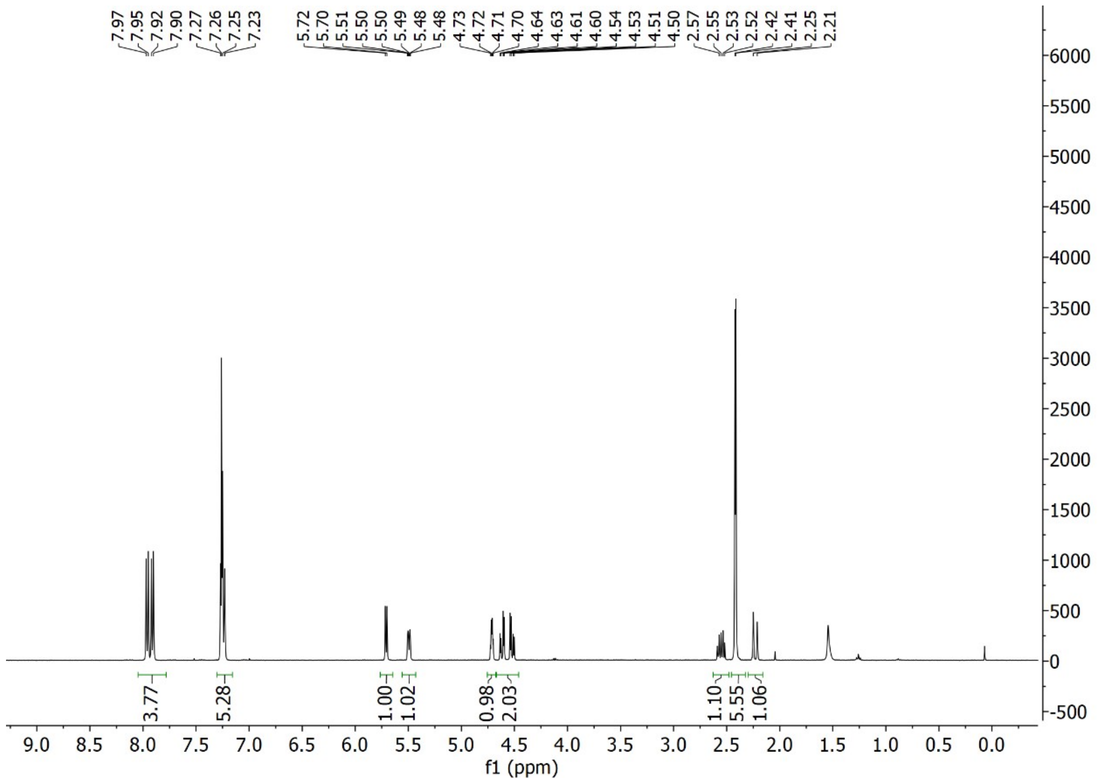

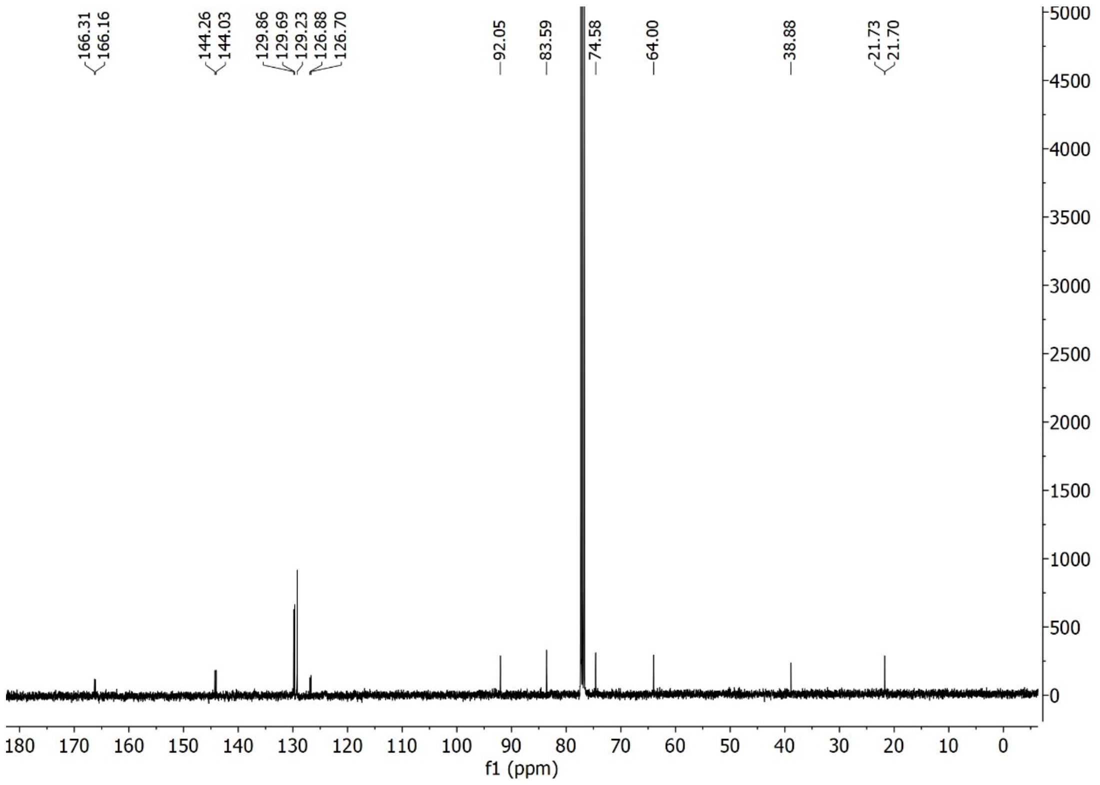

Compound 2

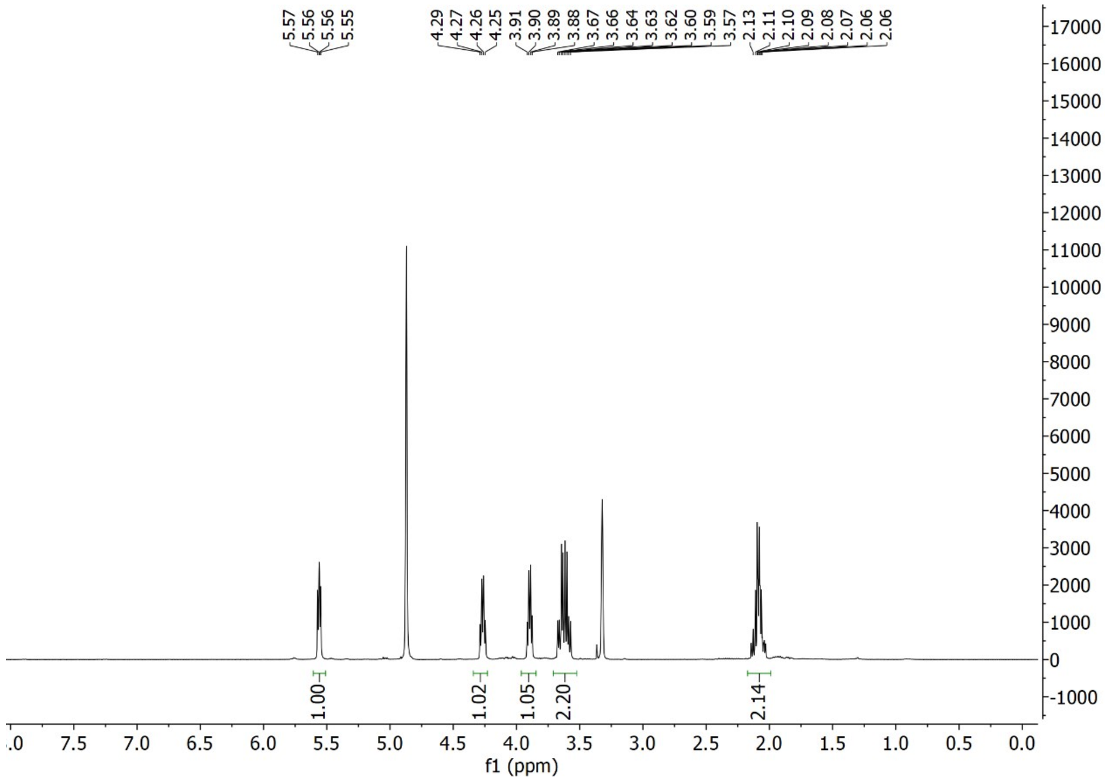
5.57
5.56
5.56
5.55
4.29
4.27
4.26
4.25
3.91
3.90
3.89
3.88
3.67
3.66
3.64
3.63
3.62
3.60
3.59
3.57
2.13
2.11
2.10
2.09
2.08
2.07
2.06
2.06
1.00
1.02
1.05
2.20
2.14
f1 (ppm)

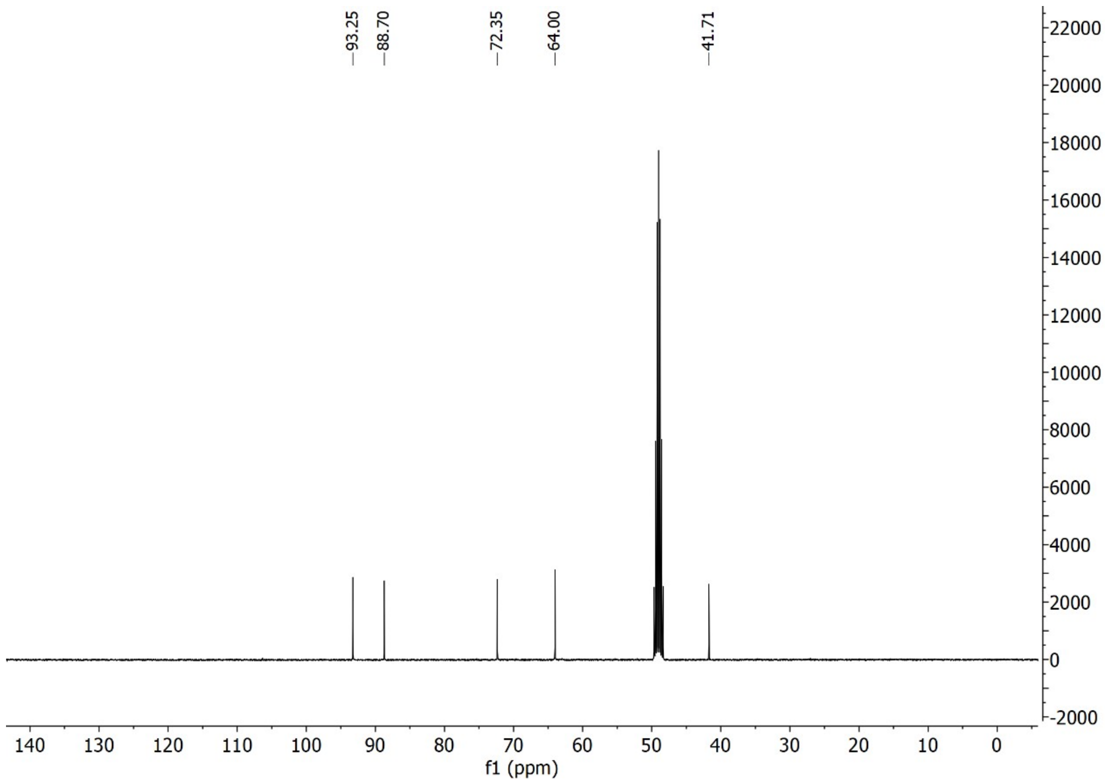
93.25
88.70
72.35
64.00
41.71
f1 (ppm)

Compound 3

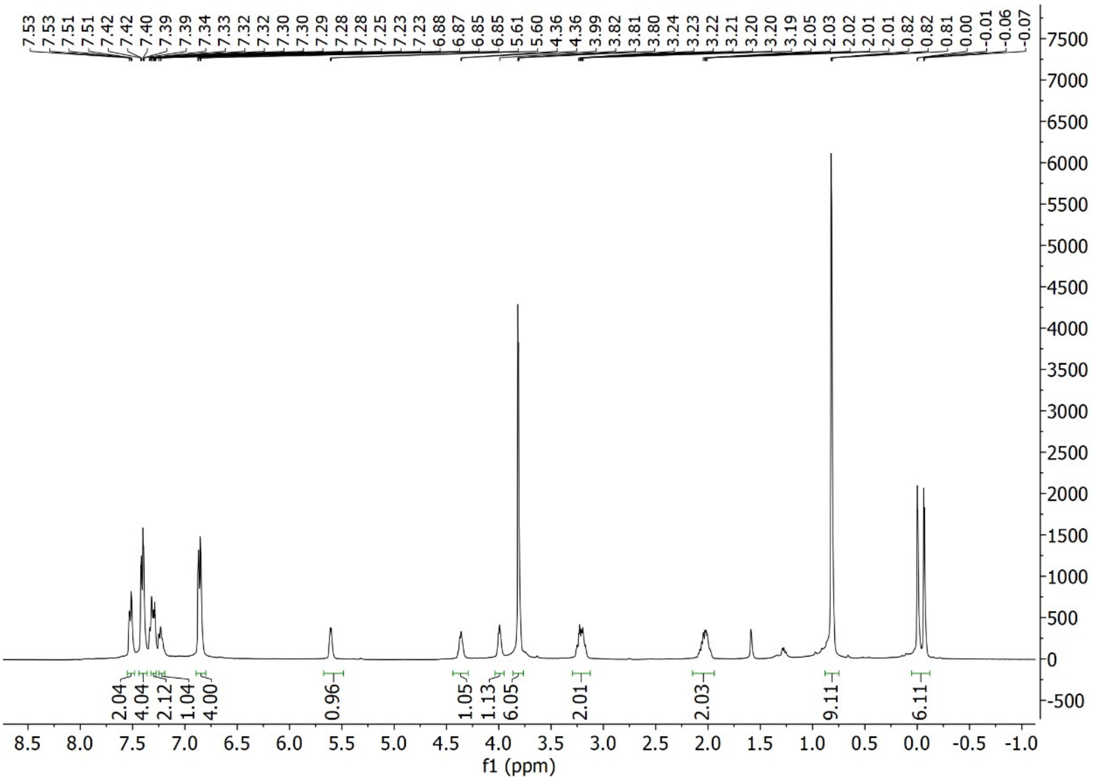
7.53
7.53
7.51
7.51
7.42
7.42
7.40
7.39
7.39
7.34
7.33
7.32
7.32
7.30
7.30
7.29
7.28
7.28
7.25
7.23
7.23
6.88
6.87
6.85
6.85
5.61
5.60
4.36
4.36
3.99
3.82
3.81
3.80
3.24
3.23
3.22
3.21
3.20
3.20
3.19
2.05
2.03
2.02
2.01
2.01
0.82
0.82
0.81
0.00
-0.01
-0.06
-0.07
2.04
4.04
2.12
1.04
4.00
0.96
1.05
1.13
6.05
2.01
2.03
9.11
6.11
f1 (ppm)

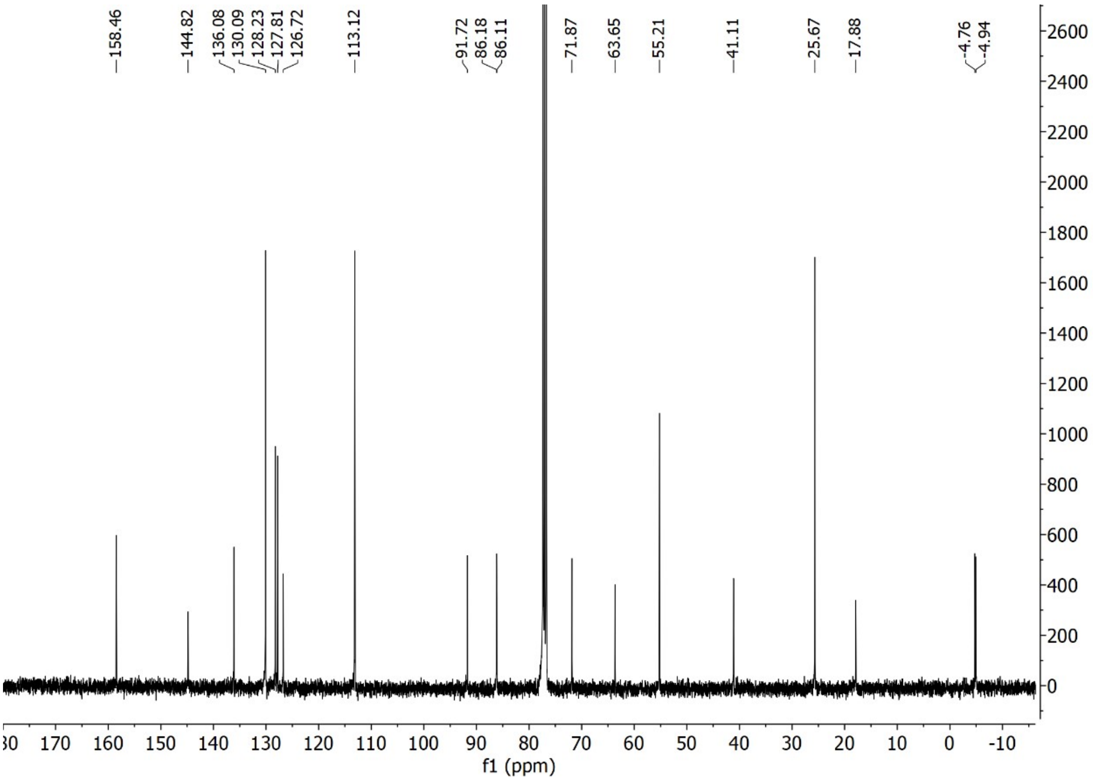
158.46
144.82
136.08
130.09
128.23
127.81
126.72
113.12
91.72
86.18
86.11
71.87
63.65
55.21
41.11
25.67
17.88
-4.76
-4.94
f1 (ppm)

Compound 4

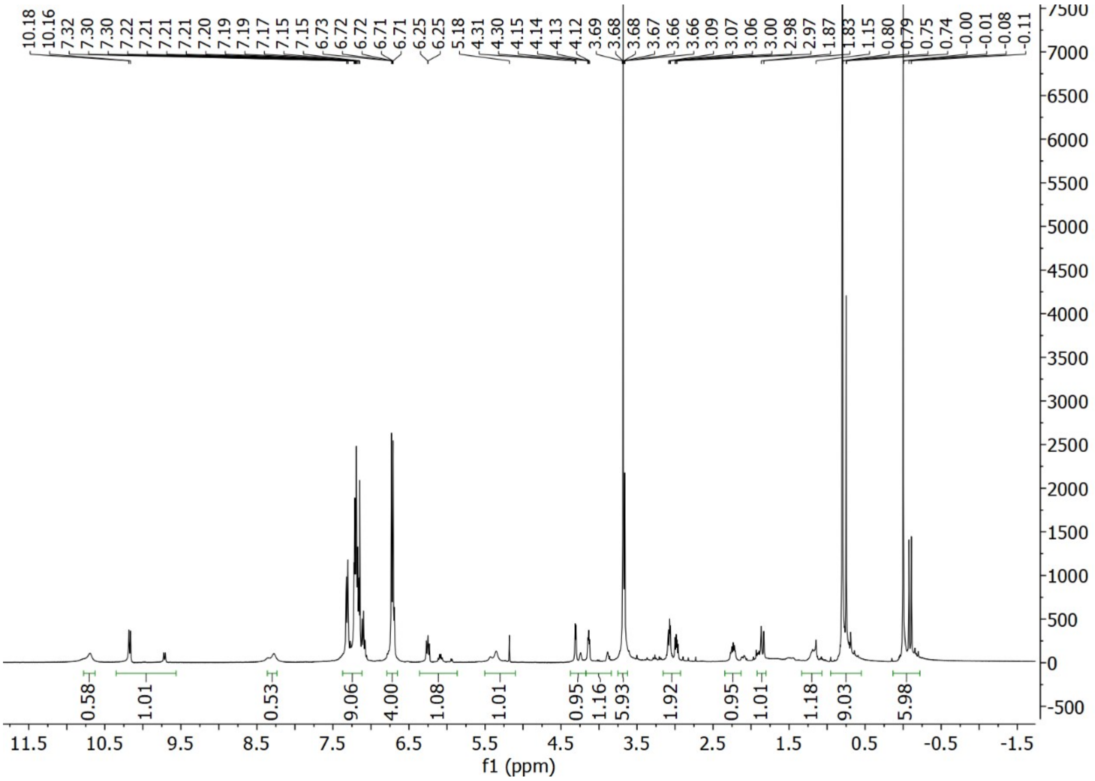

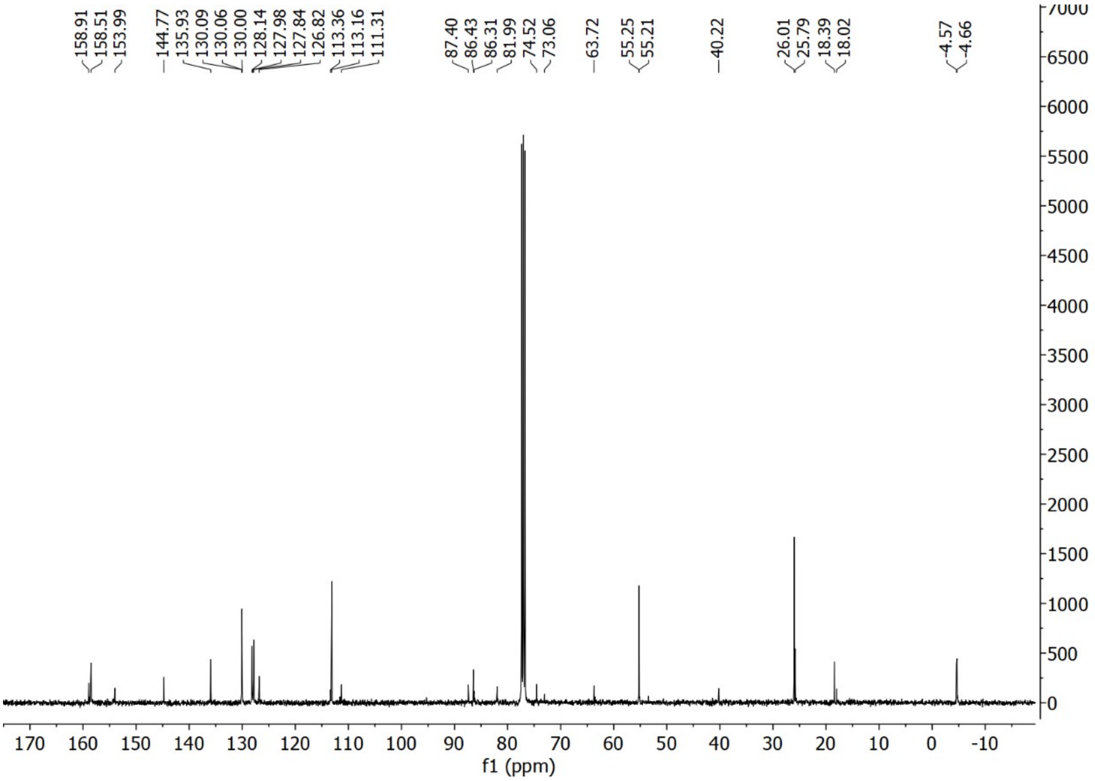

Compound 5

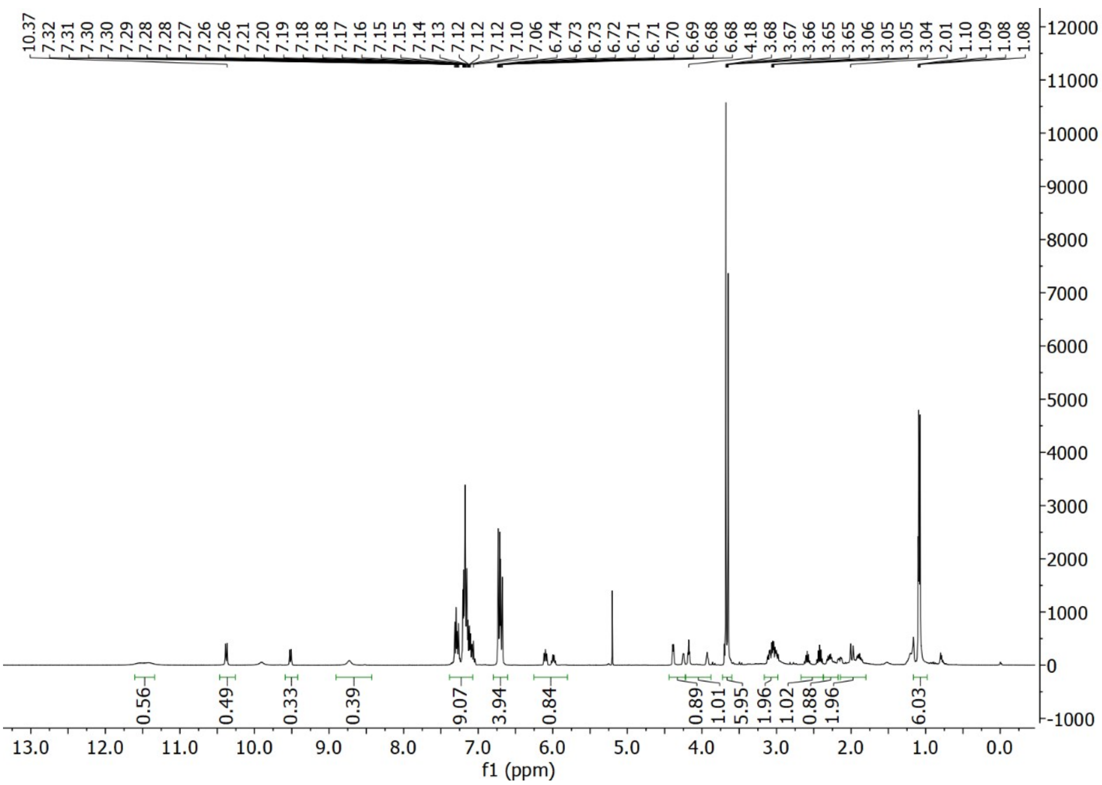

10.37 7.32 7.31 7.30 7.29 7.28 7.28 7.27 7.26 7.21 7.20 7.19 7.18 7.18 7.17 7.16 7.15 7.15 7.14 7.13 7.12 7.12 7.12 7.10 7.06 6.74 6.73 6.73 6.72 6.71 6.71 6.70 6.69 6.68 6.68 4.18 3.68 3.67 3.66 3.65 3.65 3.06 3.05 3.05 3.04 2.01 1.10 1.09 1.08 1.08
0.56 0.49 0.33 0.39 9.07 3.94 0.84 0.89 1.01 5.95 1.96 1.02 0.88 1.96 6.03
f1 (ppm)

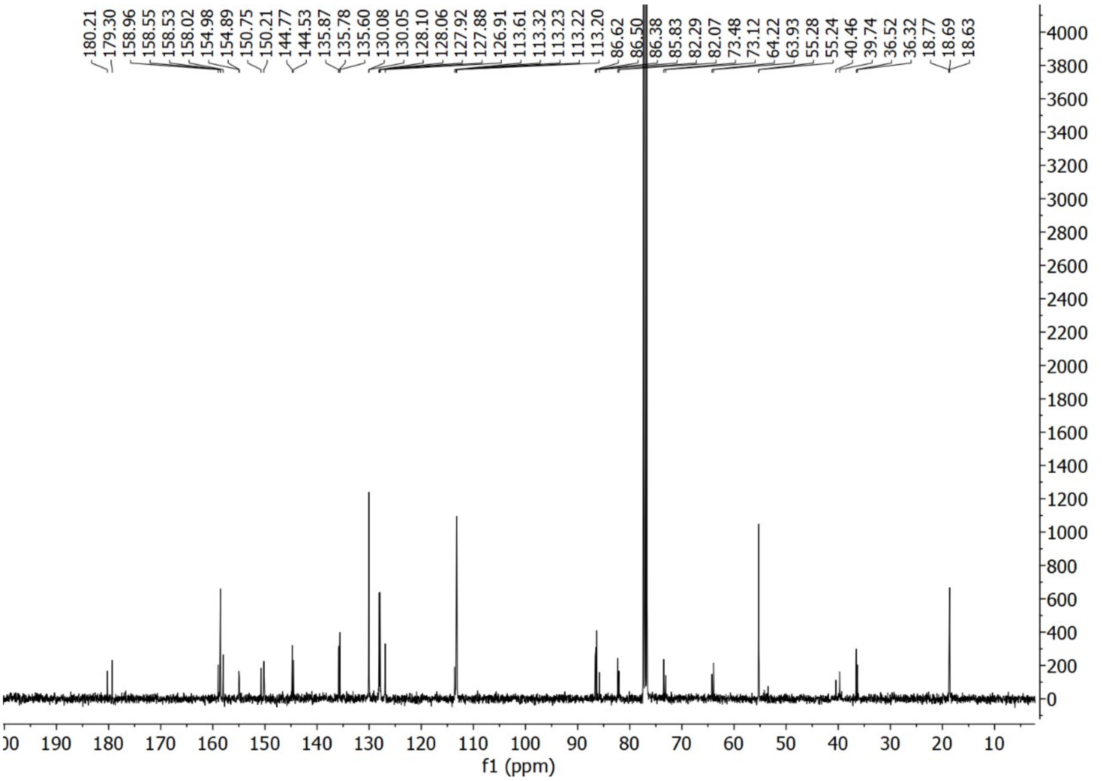

180.21 179.30 158.96 158.55 158.53 158.02 154.98 154.89 150.75 150.21 144.77 144.53 135.87 135.78 135.60 130.08 130.05 128.10 128.06 127.92 127.88 126.91 113.61 113.32 113.23 113.22 113.20 86.62 86.50 86.38 85.83 82.29 82.07 73.48 73.12 64.22 63.93 55.28 55.24 40.46 39.74 36.52 36.32 18.77 18.69 18.63
f1 (ppm)

Compound 7

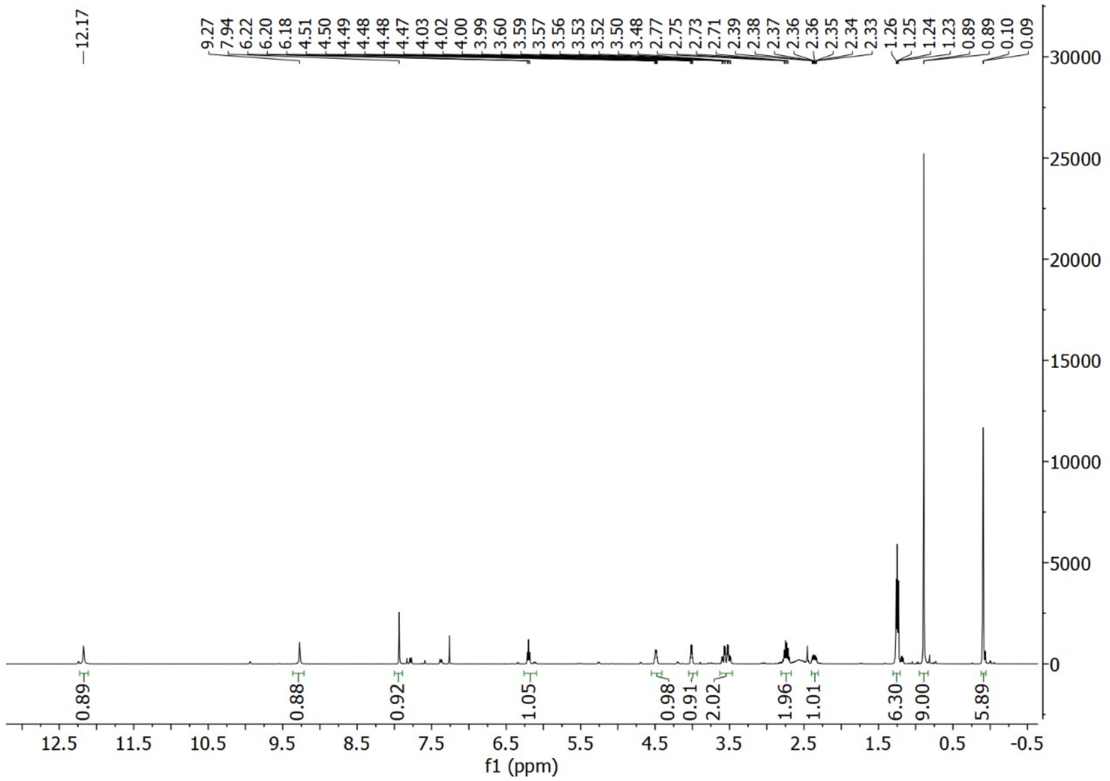
12.17
9.27
7.94
6.22
6.20
6.18
4.51
4.50
4.49
4.48
4.48
4.47
4.03
4.02
4.00
3.99
3.60
3.59
3.57
3.56
3.53
3.52
3.50
3.48
2.77
2.75
2.73
2.71
2.39
2.38
2.37
2.36
2.36
2.35
2.34
2.33
1.26
1.25
1.24
1.23
0.89
0.89
0.10
0.09
0.89
0.88
0.92
1.05
0.98
0.91
2.02
1.96
1.01
6.30
9.00
5.89
f1 (ppm)

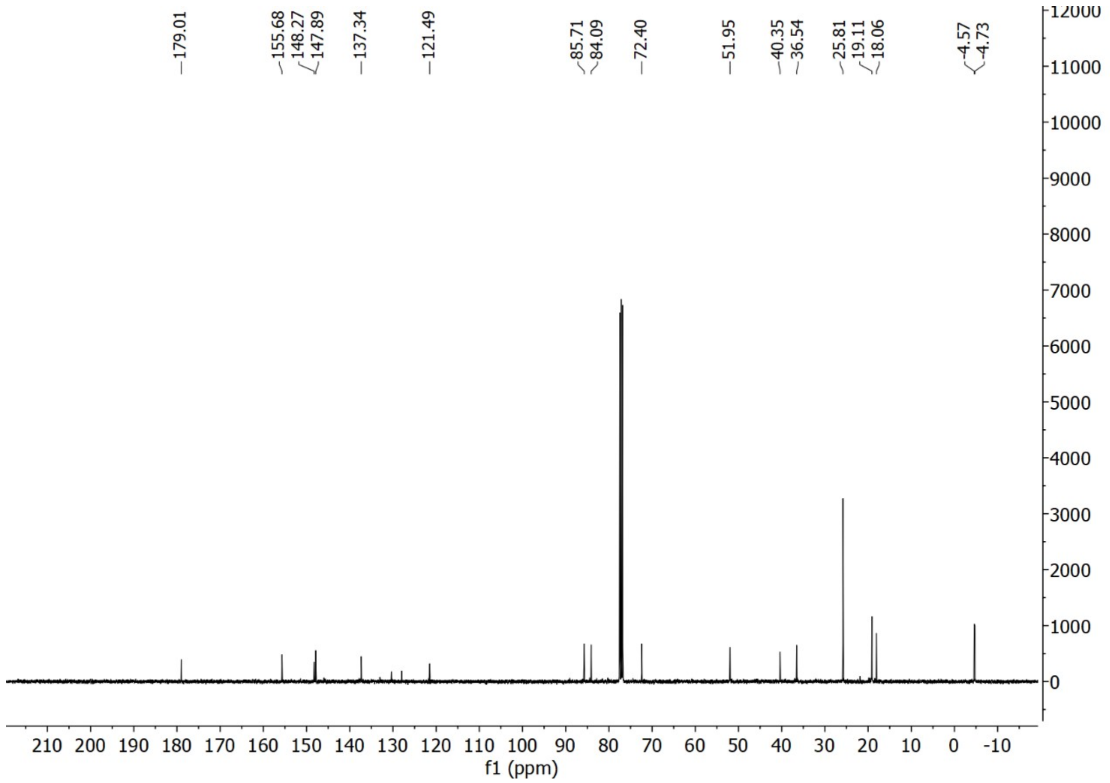
179.01
155.68
148.27
147.89
137.34
121.49
85.71
84.09
72.40
51.95
40.35
36.54
25.81
19.11
18.06
-4.57
-4.73
f1 (ppm)

Compound 8

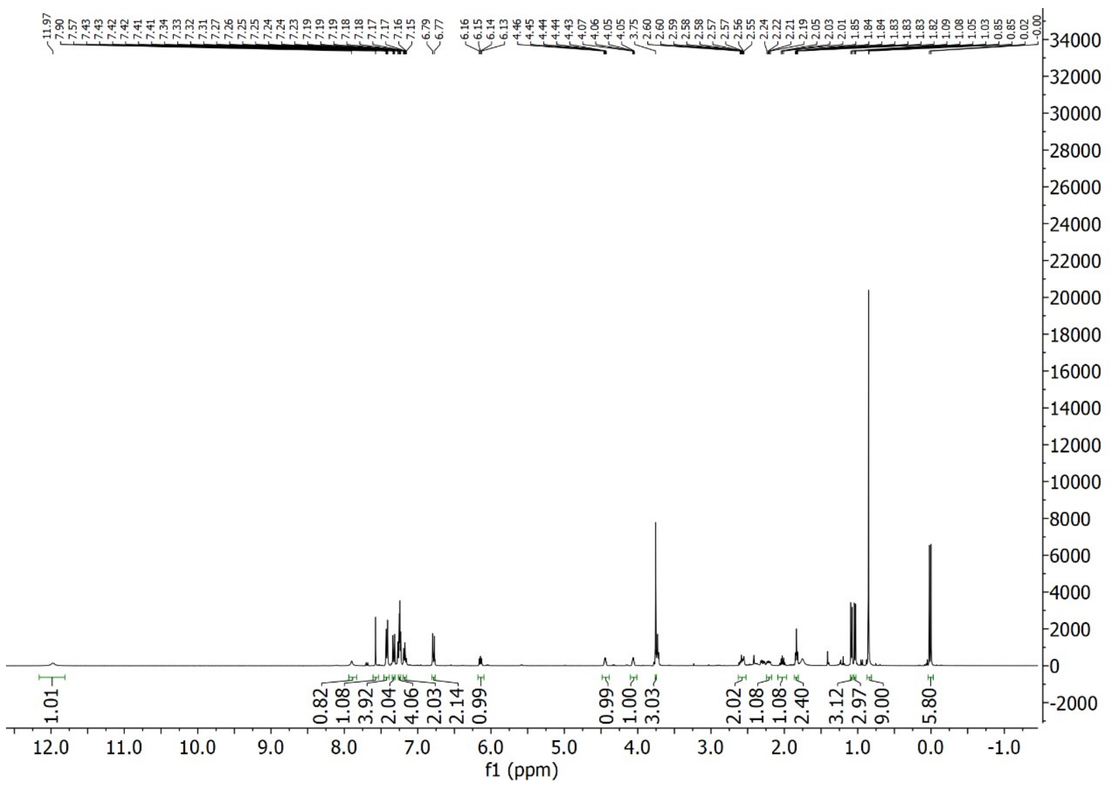

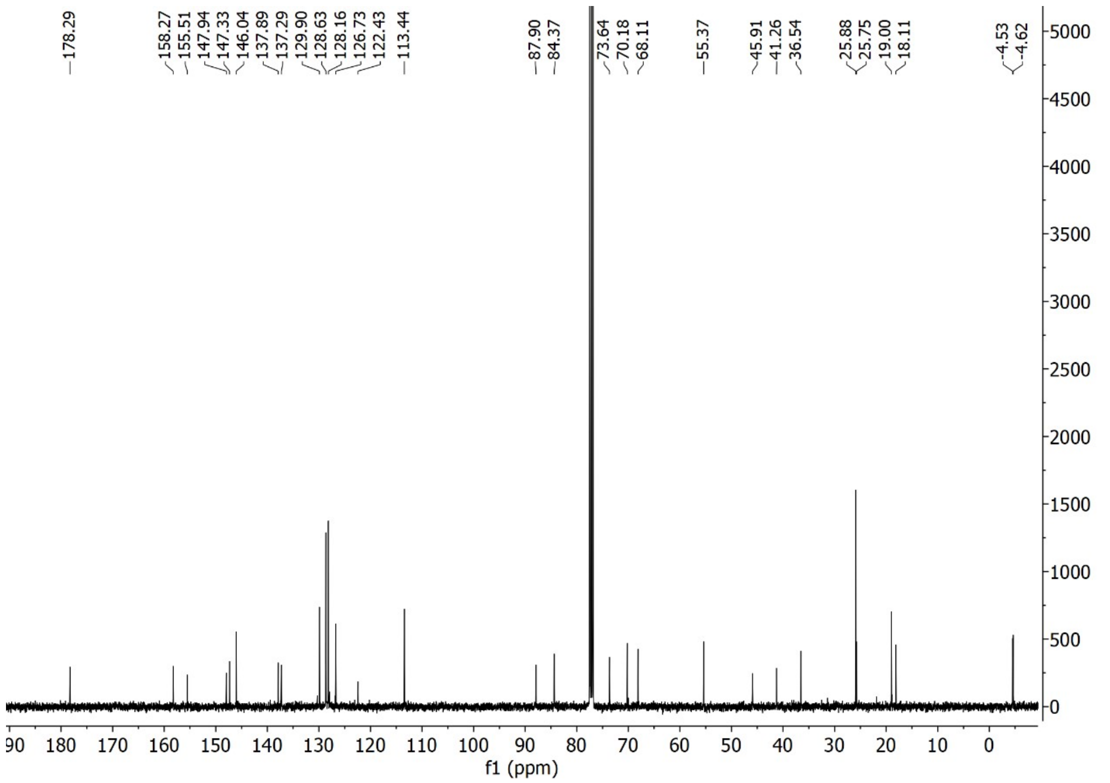

Compound 9

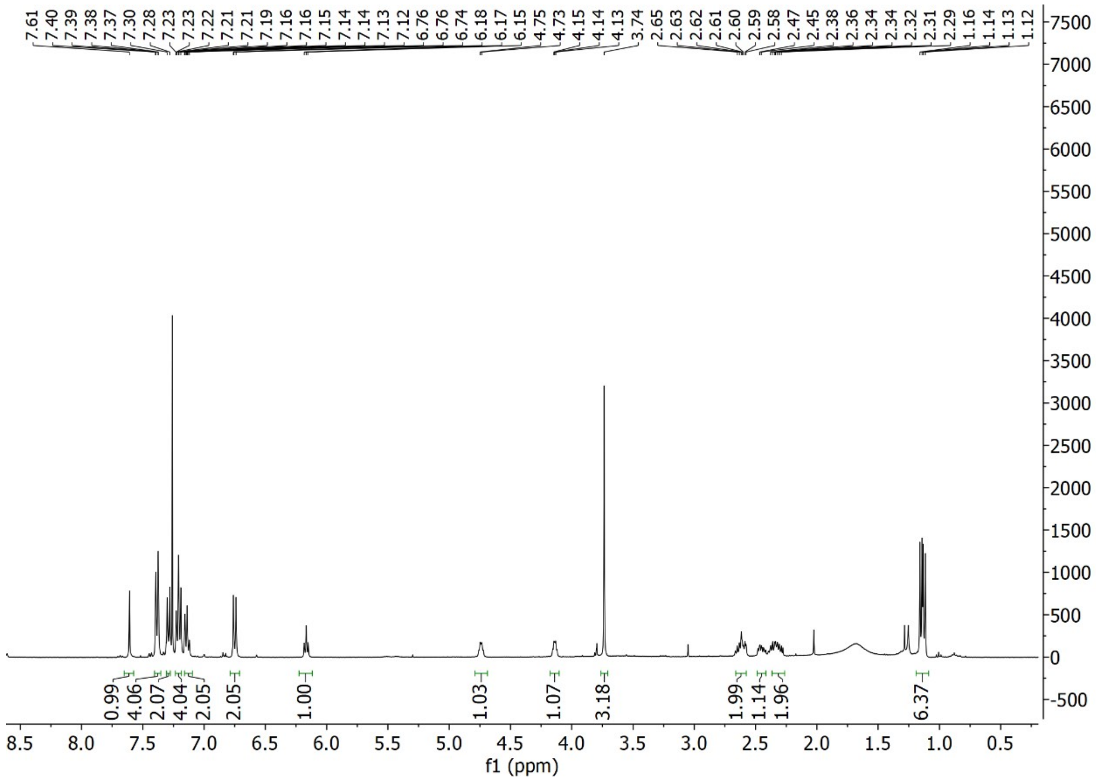

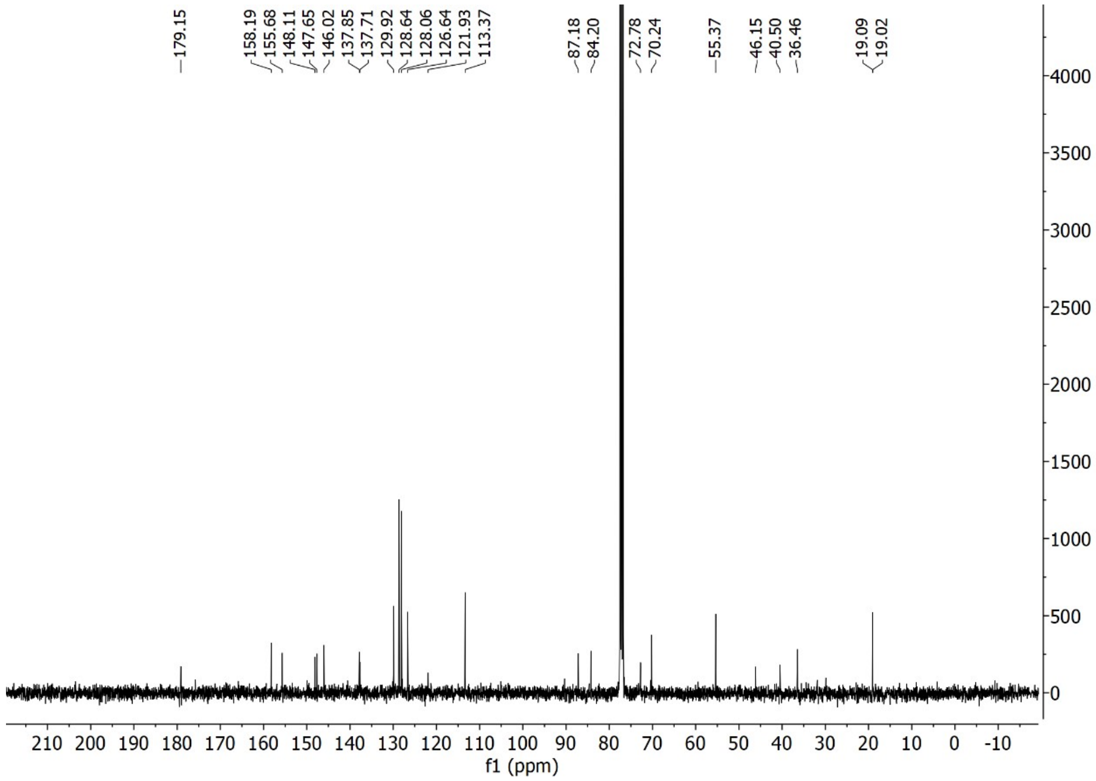

Supplement: SC-013-D1SC05648D-s001 [file SC-013-D1SC05648D-s001.pdf]
